# MCU Upregulation Overactivates Mitophagy by Promoting VDAC1 Dimerization and Ubiquitination in the Hepatotoxicity of Cadmium

**DOI:** 10.1002/advs.202203869

**Published:** 2023-01-15

**Authors:** Cong Liu, Hui‐Juan Li, Wei‐Xia Duan, Yu Duan, Qin Yu, Tian Zhang, Ya‐Pei Sun, Yuan‐Yuan Li, Yong‐Sheng Liu, Shang‐Cheng Xu

**Affiliations:** ^1^ Chongqing Key Laboratory of Prevention and Treatment for Occupational Diseases and Poisoning Chongqing 400060 P. R. China; ^2^ National Emergency Response Team for Sudden Poisoning the First Affiliated Hospital of Chongqing Medical and Pharmaceutical College Chongqing 400060 P. R. China; ^3^ Institute of Rocket Force Medicine State Key Laboratory of Trauma Burns and Combined Injury Third Military Medical University Chongqing 400038 P. R. China; ^4^ Bioengineering College of Chongqing University Chongqing 400044 P. R. China; ^5^ School of Public Health Nanjing Medical University 211166 Nanjing P. R. China

**Keywords:** cadmium, hepatotoxicity, mitochondrial calcium uniporter, mitophagy, voltage‐dependent anion‐selective channel protein 1

## Abstract

Cadmium (Cd) is a high‐risk pathogenic toxin for hepatic diseases. Excessive mitophagy is a hallmark in Cd‐induced hepatotoxicity. However, the underlying mechanism remains obscure. Mitochondrial calcium uniporter (MCU) is a key regulator for mitochondrial and cellular homeostasis. Here, Cd exposure upregulated MCU expression and increased mitochondrial Ca^2+^ uptake are found. MCU inhibition through siRNA or by Ru360 significantly attenuates Cd‐induced excessive mitophagy, thereby rescues mitochondrial dysfunction and increases hepatocyte viability. Heterozygous MCU knockout mice exhibit improved liver function, ameliorated pathological damage, less mitochondrial fragmentation, and mitophagy after Cd exposure. Mechanistically, Cd upregulates MCU expression through phosphorylation activation of cAMP‐response element binding protein at Ser133(CREB^S133^) and subsequent binding of MCU promoter at the TGAGGTCT, ACGTCA, and CTCCGTGATGTA regions, leading to increased MCU gene transcription. The upregulated MCU intensively interacts with voltage‐dependent anion‐selective channel protein 1 (VDAC1), enhances its dimerization and ubiquitination, resulting in excessive mitophagy. This study reveals a novel mechanism, through which Cd upregulates MCU to enhance mitophagy and hepatotoxicity.

## Graphical abstract

After cadmium (Cd) exposure, the cytosolic Ca^2+^‐dependent increases and activation of cAMP‐response element binding protein (CREB) orchestrally upregulates mitochondrial calcium uniporter (MCU) gene transcription, and promotes its translocation into mitochondria. The upregulated MCU in mitochondria directly interacts with voltage‐dependent anion‐selective channel protein 1 (VDAC1) and further enhances the dimerization and ubiquitination of VDAC1, which then overactivates mitophagy and leads to hepatocyte death.

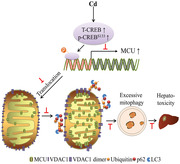

## 1 Introduction

Cadmium (Cd) is a general heavy metal pollutant widely distributed in the environment that is easily absorbed by human beings in various forms; it deposits in target tissues and has long been a threat to human health.^[^ ^1^ ^]^ The liver is a critical target organ of Cd toxicity. Epidemiological studies revealed that Cd exposure boosted nonalcoholic fatty liver disease (NAFLD), nonalcoholic steatohepatitis (NASH), hepatocellular carcinoma (HCC), hepatic steatosis and fibrosis, hepatic necroinflammation and dysfunction in humans.^[^ ^2^ ^]^ Moreover, animal studies also demonstrated that Cd exposure resulted in the destruction of liver structure, inflammation, steatosis, and fibrosis or the progression of liver cancer.^[^ ^3^ ^]^ The underlying mechanisms responsible for Cd‐induced hepatotoxicity remain obscure. Discovering novel pathogenesis for Cd‐induced hepatotoxicity would provide new independent risk factors and therapeutic strategies.

Mitochondria serve as the primary target organelles of Cd in its hepatotoxicity.^[^ ^4^ ^]^ In various disease models, Cd exposure promoted the rupture of the outer and inner mitochondrial membranes (OMM and IMM), mitochondrial swelling, and fragmentation.^[^ ^1c^ ^]^ Our previous studies revealed that overactivated mitophagy was highly involved in Cd‐induced mitochondrial dysfunction in hepatotoxicity.^[^ ^5^ ^]^ In contrast to mitophagy in healthy hepatocytes, Cd induced excessive mitophagy, resulting in massive loss of normal mitochondria, disturbance of mitochondrial metabolism, such as a decline in ATP synthesis, and overproduction of mitochondrial reactive oxygen species (MtROS), the key mediators for Cd‐induced hepatotoxicity.^[^ ^4^, ^5^ ^]^ These studies highlight that excessive mitophagy is a pivotal hallmark for Cd‐induced hepatotoxicity and suggest that targeting it could be a novel therapeutic strategy.

Homeostasis of mitochondrial Ca^2+^ maintained normal mitochondrial morphology and biological function.^[^ ^6^ ^]^ As recently reported, disturbance of this homeostasis such as persistent mitochondrial Ca^2+^ overload, promoted mitochondrial fission and mitophagy to clear damaged mitochondrial sectors.^[^ ^7^ ^]^ Mitochondrial calcium uniporter (MCU) forms a complex with its regulatory subunits and is the principle Ca^2+^ uptake channel on IMM.^[^ ^8^ ^]^ MCU has been identified as the core pore‐forming molecule of the MCU complex, which intensively regulates mitochondrial Ca^2+^ homeostasis.^[^ ^8^, ^9^ ^]^ MCU can cause liver lipid accumulation, steatosis, and metastasis of HCC cells by regulating mitochondrial Ca^2+^ signaling.^[^ ^10^ ^]^ Notably, the MCU was involved in mitochondrial quality surveillance through DRP1‐ZIP1‐mediated mitophagy to maintain the dynamics of the mitochondrial network under normal conditions.^[^ ^11^ ^]^ MCU was further found to mediate injury of neurocytes subjected to ischemia/reperfusion (IR) or lanthanum chloride (LaCl) treatment through mitophagy enhancement.^[^ ^12^ ^]^ Combined with our previous study demonstrating that Cd exposure markedly increased mitochondrial Ca^2+^, we hypothesized that MCU could be a critical molecule modulating excessive mitophagy in the hepatotoxicity of Cd.^[^ ^5a^ ^]^

Herein, we discovered that Cd exposure significantly promoted MCU expression through cAMP‐response element binding protein (CREB) and subsequently induced excessive mitophagy by increasing voltage‐dependent anion‐selective channel protein 1 (VDAC1) dimerization and ubiquitination. Our results identified MCU as a novel therapeutic target to fight against hepatotoxicity of Cd. Moreover, the upregulation of MCU could be served as a potential biomarker for Cd‐induced hepatotoxicity.

## 2 Results

### 2.1 The Expression of MCU Was Upregulated in Cd‐Induced Excessive Mitophagy in Liver Cells

Overexpression of microtubule‐associated protein light chain 3 (LC3II) and reduced expression of sequestosome 1 (SQSTM1/p62), both take part in the formation of phagophores and serve as molecular hallmarks of autophagy.^[^ ^13^ ^]^ We first confirmed Cd‐induced mitophagy and its hepatotoxicity in HepG2 liver cells.

After Cd exposure, increased LC3II and decreased p62 expression, elevated autophagy‐related genes (ATGs), accumulation of autophagosomes and autolysosomes with mitochondrial fragmentation, and increased mitophagy (monitored by indicated dye) comprehensively indicated that Cd promoted mitophagy (Figure S1A–G; Table S1, Supporting Information). Consistent with enhanced mitophagy, Cd exposure induced liver cell death in a dose‐ and time‐dependent manner (Figure S1H,I, Supporting Information). Importantly, the genetic knockdown of ATG5 or PTEN‐induced putative kinase 1 (PINK1) both obviously improved cell viability after Cd exposure (Figures S2A,B and S3A,B, Supporting Information). Furthermore, 3‐methyladenine (3‐MA), a reagent blocking the early development of phagophores and autophagosomes, significantly reduced autophagosomes and autolysosomes and efficiently improved cell viability (Figure S4A–C, Supporting Information). Although Cd exposure triggered obvious apoptosis and necrosis, the inhibition of autophagy by 3‐MA had no effects on apoptosis and necrosis caused by Cd (Figure S4D–F, Supporting Information). These data collectively verified that Cd exposure promoted the occurrence and progression of autophagy, resulting in excessive mitophagy and autophagic cell death in its hepatotoxicity.

**Figure 1:**
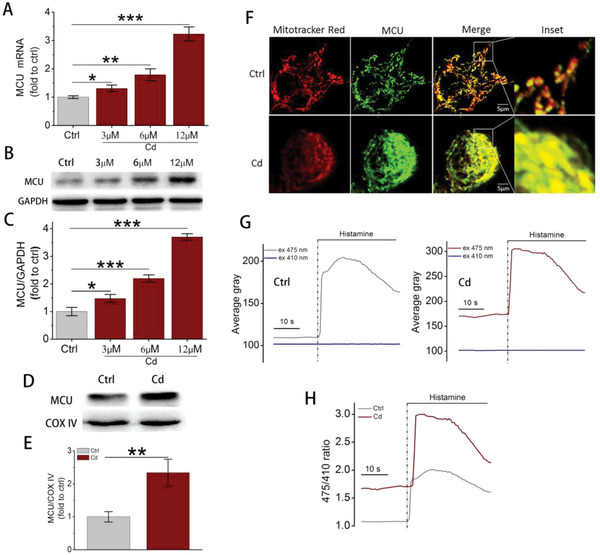
The effects of Cd exposure on MCU expression and mitochondrial Ca^2+^ uptake. A) MCU mRNA levels detected by RT‐PCR (*n* = 5). GAPDH was used as an internal control. B) Immunoblots of MCU in whole cell lysates. C) Quantification data of MCU from (B), *n =* 3. D) Immunoblots of MCU in mitochondrial lysates. COX IV was selected as the loading control. E) Quantification data of MCU from (D), *n* = 3. F) Colocalization of MCU and mitochondria analyzed by confocal microscopy. Scale bar: 5 µm. G) Average gray value tested by confocal microscopy. HepG2 cells were transfected with GCaMP6m constructs before Cd exposure to monitor mitochondrial calcium alterations. The GCaMP6m fluorescence was continuously collected at ex 475 and 410 nm simultaneously. Histamine (100 µm) was added into cells as an agonist for mitochondrial calcium. H) The 475/410 ratio calculated from (G). *p < 0.05, **p < 0.01, ***p < 0.001.

To determine the molecular mechanism by which Cd induced excessive mitophagy, we focused on MCU and its complex. RNA‐seq showed that Cd exposure promoted the gene transcription of MCU (Figure S6A; Table S2, Supporting Information). In line with the RNA‐seq results, RT‐PCR analysis also demonstrated that 3, 6, and 12 µm Cd exposure for 12 h increased MCU mRNA levels in a dose‐dependent manner, which was further confirmed by elevated protein expression (**Figure** **1**A–C). Moreover, immunoblots of mitochondrial lysates and immunofluorescent staining both demonstrated that Cd exposure promoted the recruitment of overexpressed MCU to mitochondria (Figure 1D–F). The alteration of [Ca^2+^]_m_ was monitored by transfecting cells with GCaMP6m constructs. [Ca^2+^]_m_ probes revealed an increased [Ca^2+^]_m_ signal after 12 µM Cd exposure (Figure 1G,H). Regarding regulatory proteins for MCU, RNA‐seq showed that Cd exposure promoted the gene transcription of essential MCU regulator (EMRE) and mitochondrial calcium uptake (MICU)2, and reduced MICU1 while did not affect MCU‐dominant negative β‐subunit (MCUb) (Figure S6A; Table S2, Supporting Information). Additionally, the immunoblot data showed that Cd exposure significantly increased the protein expression of EMRE but did not alter MCUb, MICU1, and MICU2, which mostly agreed with the RNA‐seq results (Figure S6B,C, Supporting Information). These findings indicated that Cd exposure induced MCU upregulation and [Ca^2+^]_m_ overload.

### 2.2 Inhibition of MCU Alleviated Autophagic Cell Death Induced by Cd Exposure

**Figure 2:**
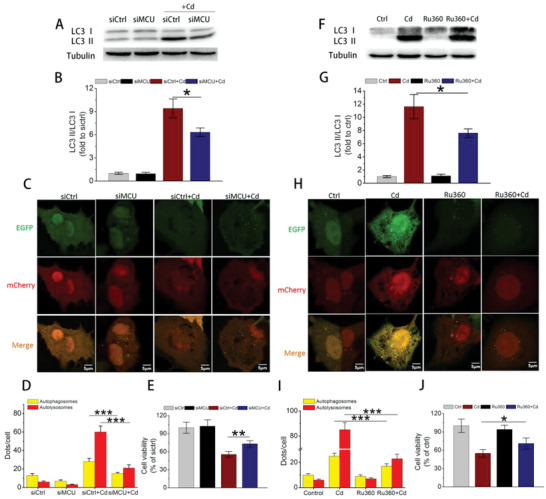
The effects of MCU inhibition on Cd‐induced autophagic cell death. A) Immunoblots and B) quantification of LC3 II/I for evaluating the effect of MCU knockdown on autophagy (*n* = 3). The siMCU was transfected into cells 48 h before Cd exposure. C) Cells were infected with EGFP‐mCherry‐LC3 adenovirus, and the effects of siMCU on autophagic flux were monitored. Representative confocal images were shown. D) Quantification of autophagosomes and autolysosomes (*n* = 20). E) Cell viability change in cells treated with siMCU and Cd. F–J) The effects of Ru360 (5 µm, 2 h before Cd exposure) on autophagy were further evaluated using methods similar to those of siMCU. *p < 0.05, **p < 0.01, ***p < 0.001.

To investigate whether MCU upregulation overactivated mitophagy, we downregulated MCU expression by specific siRNA (siMCU) or inhibited its function by Ru360 before Cd exposure (Figure S2C, Supporting Information). As a result, siMCU successfully decreased LC3II expression (**Figure** **2**A,B) and reduced autophagosomes and autolysosomes in Cd‐treated cells (Figure 2C,D), then improved cell viability (Figure 2E). Consistently, Ru360, a specific mitochondrial calcium uptake inhibitor, yielded similar effects with those of siMCU (Figure 2F–J). In primary cultured hepatocytes isolated from MCU^+/+^ (WT) and MCU^−/−^ (KO) mice, MCU knockout effectively decreased LC3 II expression, inhibited the translocation of PINK1 and Parkin into mitochondria and prevented the occurrence of mitophagy (Figures S7A–C and S8, Supporting Information). These data indicated that Cd induced excessive mitophagy and hepatotoxicity through upregulation of MCU.

### 2.3 MCU Inhibition Improved Mitochondrial Dysfunction Caused by Cd Exposure

**Figure 3:**
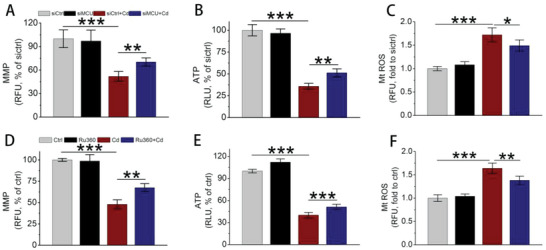
The effects of siMCU and Ru360 on Cd‐induced mitochondrial dysfunction. A–C) Effects of siMCU on (A) MMP detected by JC‐1, (B) ATP content, and (C) mitochondrial ROS production tested by MitoTracker Red CM‐H_2_XRos (*n* = 5). D–F) Effects of Ru360 on (D) MMP, (E) ATP, and (F) mitochondrial ROS by the methods used in (A)–(C), respectively (*n* = 5). MMP: mitochondrial membrane potential. *p < 0.05, **p < 0.01, ***p < 0.001.

Next, the effects of MCU upregulation on mitochondrial function were evaluated. The data showed that Cd exposure induced significant mitochondrial membrane potential (MMP) reduction, ATP decline, and mitochondrial ROS (MtROS) overproduction (**Figure** **3**A–F). Notably, MCU knockdown by siMCU effectively restored MMP, increased ATP, and reduced MtROS (Figure 3A–C). In line with the results of siMCU, Ru360 was also found to be effective in improving mitochondrial dysfunction (Figure 3D–F). These data demonstrated that overexpressed MCU accelerated mitochondrial dysfunction, one outcome of excessive mitophagy.

### 2.4 MCU Knockdown Attenuated Excessive Mitophagy and Liver Injury In Vivo

**Figure 4:**
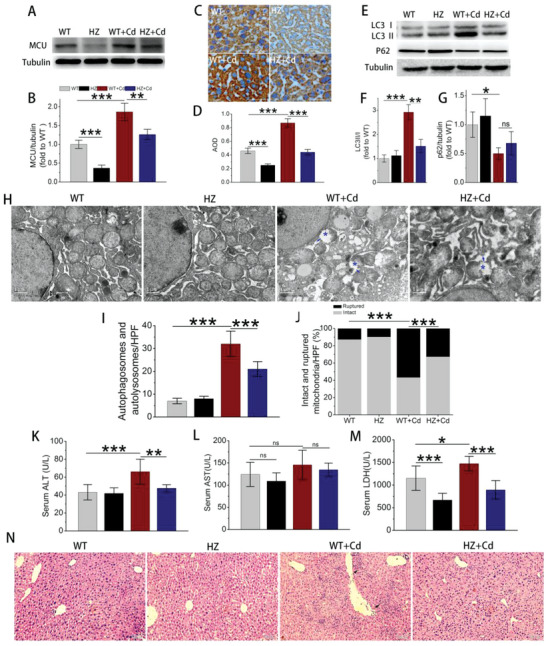
Evaluation of excessive mitophagy and hepatotoxicity in heterogeneous MCU KO mice exposed to Cd. The MCU^+/+^ and MCU^+/−^ CD1 mice were exposed to saline or Cd for 7 consecutive days and anesthetized for blood or liver yielding (*n* = 10). A) Immunoblots of MCU in fresh liver lysates. B) Quantification of MCU from (A), *n* = 3. C) Immunohistochemistry (IHC) staining of MCU in liver tissues. 400× magnification images were taken and 10 fields each group were randomly chosen for analysis. D) Average optical density calculated from (C). E) Immunoblots of LC3 ǁ/ǀ and p62 in fresh liver lysates. F) Quantification of LC3 ǁ/ǀ and G) p62 from (E), *n* = 3. H) TEM images of liver tissue. Blue asterisks indicate impaired mitochondria, and blue arrows indicate ruptured mitochondrial membranes. Scale bar: 1 µm. I) Total autophagosomes and autolysosomes counted from TEM images (*n* = 10). J) Percentages of intact and ruptured mitochondria calculated from TEM images. Chi‐square test was used for comparison between groups. K) Serum ALT, L) AST, and M) LDH were detected using a biochemistry analyzer (*n* = 5–6). N) HE staining of liver tissue. Magnification, 200×. Scale bar: 100 µm. *p < 0.05, **p < 0.01, ***p < 0.001. ns, no significance.

Wild‐type (WT, MCU^+/+^) and heterozygous (HZ, MCU^+/−^) mice were harvested to verify whether MCU upregulation overactivated mitophagy in vivo (Figure S7A–C, Supporting Information). The MCU knockout (KO, MCU^−/−^) mice presented a lower body weight than WT and HZ mice, similar to that of WT mice treated with Cd (Figure S7D,E, Supporting Information). Therefore, we utilized HZ, whose MCU expression was nearly 50% that of WT. The immunoblots and immunohistochemical staining assays revealed that Cd exposure upregulated MCU expression in both WT and HZ livers (**Figure** **4**A–D). Additionally, Cd exposure promoted LC3II formation and inversely decreased p62 expression, the former was efficiently rescued in HZ (Figure 4E–G). Transmission electronic microscopy (TEM) showed that Cd exposure resulted in abundant formation of autophagosomes/autolysosomes surrounding the mitochondria and largely loss of mitochondrial cristae and breakage of the OMM/IMM (Figure 4H). In line with the results of immunoblots, the total number of autophagosomes/autolysosomes and the proportion of ruptured mitochondria (rupture of OMM/IMM) were both significantly reduced in hepatocytes from HZ with Cd exposure (Figure 4I,J). Next, we further assessed the alteration of liver function. The concentrations of alanine transaminase (ALT) and lactate dehydrogenase (LDH) in WT mice were sharply elevated, while the aspartate transaminase (AST) level showed an increasing trend after Cd exposure (Figure 4K–M). Importantly, the overproduction of ALT and LDH was markedly inhibited in Cd‐treated HZ mice (Figure 4K–M). Consistent with the results of liver function, Cd exposure destroyed the classic structure of the liver lobule, resulting in tissue edema and extensive penetration of inflammatory cells, which were efficiently attenuated in HZ (Figure 4N). These in vivo data collectively demonstrated that the upregulation of MCU triggered by Cd exposure led to excessive mitophagy, thereby promoting the hepatotoxicity of Cd in vivo.

### 2.5 CREB Upregulated MCU Expression by Binding to the MCU Promoter to Enhance Gene Transcription

**Figure 5:**
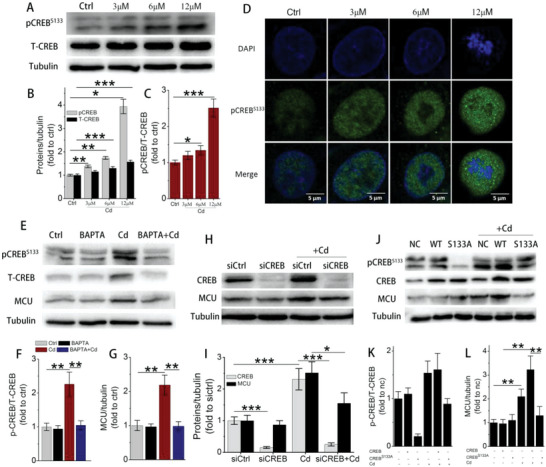
The regulatory effect of CREB on MCU expression. A) Immunoblots and B) quantitative analysis of pCREB^S133^, T‐CREB, and C) their ratio in cell lysates after 3, 6, and 12 µm Cd exposure (*n* = 3). D) Representative confocal images of pCREB^S133^ immunostaining. The nucleus was stained with DAPI (blue). E) Immunoblots and F) quantitative analysis of pCREB^S133^/T‐CREB ratio, and G) MCU in cell lysates (*n* = 3). H) Immunoblots and I) quantitative analysis of T‐CREB and MCU for evaluating the effect of CREB on MCU expression (*n* = 3). J) Immunoblots and K) quantitative analysis of pCREB^S133^/T‐CREB ratio and L) MCU for evaluating the effect of CREB mutation at Ser133 on MCU expression (*n* = 3). *p < 0.05, **p < 0.01, ***p < 0.001.

To explore the potential upstream regulatory mechanism for MCU elevation, we focused on CREB, a critical Ca^2+^‐ sensitive transcription factor in liver.^[^ ^14^ ^]^ Upon immunoblotting, it was found that phosphorylated CREB at Ser133 (pCREB^S133^), total CREB (T‐CREB), and their ratio (pCREB^S133^/T‐CREB) increased in a dose‐dependent manner after 3, 6, and 12 µm Cd exposure for 12 h (**Figure** **5**A–C). Following immunoblotting, confocal microscopy revealed that Cd exposure induced the gradual accumulation of pCREB^S133^ in the hepatocellular nucleus, consistent with its protein expression (Figure 5D; Figure S10A,B, Supporting Information). Interestingly, the accumulation of pCREB^S133^ in nucleus was positively correlated with upregulation of MCU (Figure S10C, Supporting Information). As an important Ca^2+^‐response nuclear transcription factor, we next utilized BAPTA‐AM to chelate cytosolic Ca^2+^ to explore the alteration of CREB and MCU expression. The immunoblotting data presented here revealed that BAPTA‐AM reduced the expression of pCREB^S133^ and T‐CREB and their ratio and reduced the upregulation of MCU (Figure 5E–G). Concurrently, BAPTA‐AM was also effective in attenuating autophagic cell death (Figure S11A–E, Supporting Information), which was further improved by siMCU (Figure S11F,G, Supporting Information). Finally, we investigated whether CREB regulated MCU expression in hepatocytes, and the results showed that CREB knockdown (siCREB; Figure S2D, Supporting Information) or its mutation at the Ser133 site (pCREB^S133A^) both successfully downregulated MCU upregulation (Figure 5H–L). These data innovatively revealed that CREB positively regulated MCU expression in a cytosolic Ca^2+^‐dependent manner in hepatocytes.

**Figure 6:**
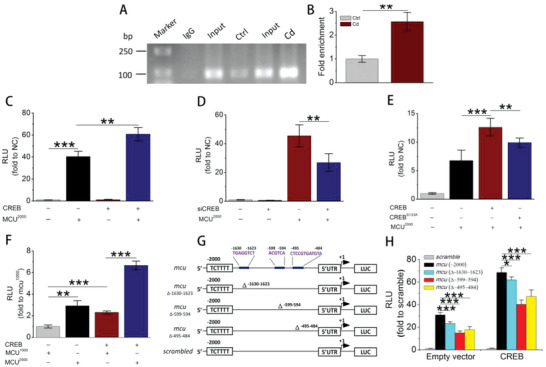
Transcriptional regulation of CREB on MCU. A) Agarose gel electrophoresis for ChIP‐PCR products. HepG2 cells were treated with or without 12 µm Cd for 12 h, and the protein–DNA complex were harvested for ChIP assay. B) Relative enrichment of pCREB^S133^ on MCU promoter (*n* = 3). C) Activation of the MCU promoter (MCU^2000^) with empty vector (basal condition, served as a negative control) or CREB (WT) plasmid transfection (*n* = 5). The RLU data are expressed as the fold change compared to the negative control (NC). D) RLU values normalized to the NC group after CREB knockdown (*n* = 5). E) RLU values normalized to the NC group after CREB phosphorylation mutation at Ser133 site (CREB^S133A^), *n* = 5. F) Comparison of MCU promoter (MCU^1000^ and MCU^2000^) activity under basal conditions or CREB treatment. Data were normalized to the MCU^1000^ group (*n* = 5). G) Schematic diagram showing three different mutation sites in the MCU 2000 bp promoter and their relative locations. H) Cells were transfected with empty vector or the indicated plasmids, including three mutated firefly luciferase reporter gene plasmids as stated in (G) and CREB (WT). The RLU values were obtained and normalized to scramble (*n* = 5). From (C) to (H), the dual luciferase reporter assay was performed on HEK293T cells with four types of plasmid transfection, including the firefly luciferase plasmids (MCU^1000^, MCU^2000^, and its mutations), the transcription factor (CREB and CREB^S133A^), the Renilla luciferase plasmids and the corresponding empty vectors. *p < 0.05, **p < 0.01, ***p < 0.001.

Next, we further investigated how CREB regulates MCU gene expression. The ChIP assay demonstrated that pCREB^S133^ could bind to MCU promoter that was enhanced by Cd exposure. It implied that CREB regulated MCU gene transcription (**Figure** **6**A,B). In HEK293T cells, after MCU^2000^ transfection, the luciferase activity, expressed as relative light units (RLU), increased significantly under basal conditions and continued to increase after exogenous CREB (wild type) transfection (Figure 6C). Conversely, knockdown and mutation at Ser133 of CREB both distinctly decreased the activity of MCU^2000^ (Figure 6D,E), which agreed with the former immunoblot results. Then, the luciferase activity levels of MCU^1000^ and MCU^2000^ were compared in the presence or absence of CREB. The RLU increased by approximately two fold in the MCU^2000^ group compared to that of MCU^1000^ in the absence or presence of CREB transfection, implying the existence of CREB‐binding sites at both the 0 to −1000 and −1000  to −2000 bp regions on the MCU promoter (Figure 6F). To explore potential binding sites, three truncated mutants of the MCU promoter, TGAGGTCT (‐1630‐1623), ACGTCA (‐599‐594), and CTCCGTGATGTA (‐495‐484), were comprehensively predicted by the indicated method (Tables S4 and S5, Supporting Information; Figure 6G). The RLUs of these three mutants were detected and analyzed by comparison with that of MCU^2000^. Under basal conditions (transfection with the empty CREB vector), the RLU of each mutant was obviously lower than that of MCU^2000^. Although CREB markedly increased the RLUs of these mutants, similar results were obtained (Figure 6H). Therefore, we revealed the TGAGGTCT (‐1630‐1623), ACGTCA (‐599‐594), and CTCCGTGATGTA (‐495‐484) sites as novel target sequences of CREB on the MCU promoter. These results implied that CREB could extensively regulate MCU expression by binding to multiple target sequences on the MCU promoter.

### 2.6 MCU Interacted with VDAC1 and Enhanced Its Dimerization after Cd Exposure

The mitochondrial permeability transition pore (mPTP) is involved in the initiation of mitophagy.^[^ ^15^ ^]^ To explore whether MCU upregulation‐induced activation of excessive mitophagy was mPTP dependent, we preliminarily assessed the opening of mPTP and its role in Cd‐induced autophagic cell death. An obvious reduction in fluorescence intensity was observed in cells exposed to 6 and 12 µm Cd for 12 h, indicating Cd‐induced mPTP opening (Figure S12A, Supporting Information). This was further proved by significant MMP reduction (Figure S12B, Supporting Information) and mitochondrial fragmentation (Figure S12C, Supporting Information). Cyclophilin D (CypD) is an essential component of mPTP, and its effect on mPTP opening was verified by siCypD and cyclosporine (CSA), a classical CypD inhibitor (Figures S2E and S13A,D,E, Supporting Information). Although Cd exposure increased the expression of CypD, both siCypD and CSA failed to alleviate autophagic cell death caused by Cd (Figure S14A–F, Supporting Information). Moreover, inhibition of another two mPTP components including ATP synthase (ATPase) and adenine nucleotide translocator (ANT2) also failed to alleviate Cd‐induced autophgic cell death (Figures S2F,G, S13B–E, and S14G–L, Supporting Information). These data comprehensively demonstrated that these classic mPTP components did not contribute to MCU upregulation‐induced excessive mitophagy and autophagic cell death under Cd hepatotoxicity.

**Figure 7:**
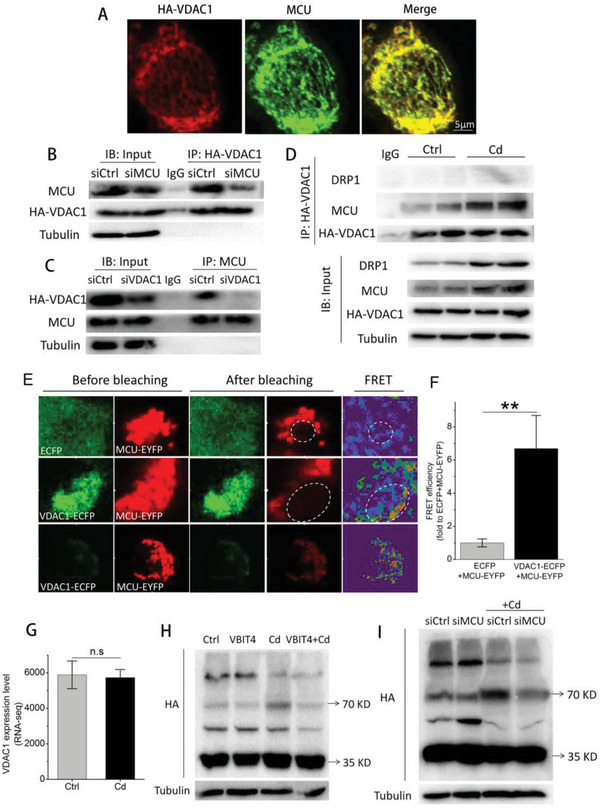
Upregulated MCU promoted VDAC1 dimerization through their physical interaction. A) Confocal images indicating mitochondrial colocalization between MCU and VDAC1 tagged with HA. B,C) Immunoblots for coimmunoprecipitation of MCU and HA‐VDAC1 in cell lysates without Cd exposure. (B) HA‐VDAC1 or (C) MCU primary antibody was used to pull down proteins binding to VDAC1 or MCU in cells treated with siMCU or siVDAC1, respectively. D) Immunoblots for coimmunoprecipitation of MCU, HA‐VDAC1, and DRP1 in cell lysates after Cd exposure. HA‐VDAC1 primary antibody was utilized to pull down proteins binding to VDAC1 in cells exposed to Cd or not exposed. E) FRET assay verified the interaction between MCU and VDAC1. The VDAC1‐ECFP and MCU‐EYFP plasmids were constructed and transfected into HepG2 cells, and the FRET was performed by photobleaching the EYFP (acceptor) on mitochondria of living HepG2 cells. The middle panel and the bottom panel indicated the FRET after total bleaching and partial bleaching of EYFP, respectively. An increased fluorescence of ECFP (donor) was observed after bleaching. F) Quantification of FRET efficiency after total bleaching (*n* = 5). The value was normalized to ECFP+MCU‐EYFP. G) RNA‐seq of VDAC1 in cells treated with or without 12 µm Cd for 12 h (*n* = 4). H,I) The effect of VBIT4 (H) or siMCU (I) on VDAC1 dimerization. Tubulin served as the loading control in these assays. **p < 0.01. ns, no significance.

To further dig out the underlying molecular mechanisms for MCU upregulation‐induced excessive mitophagy, we moved toVDAC1, an important channel protein localized at the OMM that plays a critical role in mitochondrial metabolism.^[^ ^16^ ^]^ Confocal microscopy showed a close spatial colocalization between VDAC1 and MCU (**Figure** **7**A). Importantly, coimmunoprecipitation (CoIP) using a specific anti‐HA or MCU antibody after MCU or VDAC1 knockdown revealed the physical interaction between MCU and VDAC1 (Figure 7B,C). Dynamin‐related protein 1 (DRP1) plays an important role in inducing mitochondrial fragmentation before the removal of damaged mitochondrial fractions.^[^ ^5^, ^17^ ^]^ The immunoblot results showed that Cd exposure increased the expression levels of MCU and DRP1 but not HA‐VDAC1, which was further evidenced by RNA‐seq of VDAC1 (Figure 7D,G). Moreover, the CoIP results demonstrated an elevated interaction between MCU and HA‐VDAC1 but not DRP1 (Figure 7D). Importantly, by fluorescence resonance energy transfer (FRET) assay in living HepG2 cells, we revealed that MCU could directly interact with VDAC1 (Figure 7E,F). Further, by mutating the intermembrane space (IMS) domain (aa256‐266) and transmembrane (TM) domain (aa234‐283) of MCU without affecting their mitochondrial localization, we found their interaction still existed (Figure S15A–C, Supporting Information). These data collectively confirmed that MCU directly interacted with VDAC1, and binding domain did not locate at the IMS domain and TM domain.

**Figure 8:**
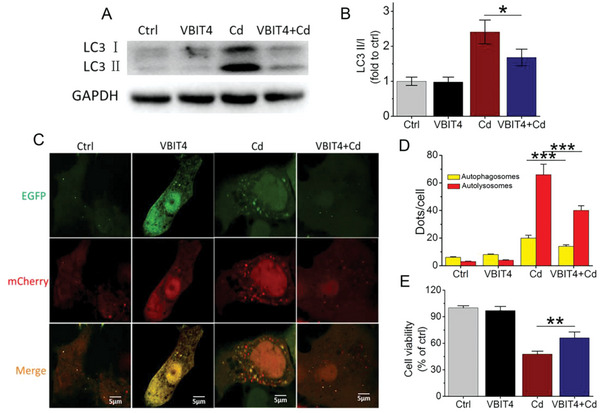
Inhibition of VDAC1 attenuated autophagic cell death caused by Cd. A) Immunoblots of LC3 ǁ/ǀ from lysates pretreated with VBIT4 for 48 h and then exposed to 12 µm Cd for 12 h. B) Quantification of LC3 ǁ/ǀ from (A), *n* = 3. C) Alternation of autophagic flux after VBIT4 treatment. Cells were treated as previously described. Scale bar: 5 µm. D) Quantification of autophagosomes and autolysomes (*n* = 20). E) Cell viability after VBIT4 treatment (*n* = 5). *p < 0.05, **p < 0.01, ***p < 0.001.

By intercellular protein crosslinking technique, we next revealed the increased dimerization of VDAC1 after Cd exposure (Figure 7H,I). VBIT4, an inhibitor of VDAC1,^[^ ^18^ ^]^ inhibited the dimerization of VDAC1 (Figure 7H). Surprisingly, the downregulation of MCU by siMCU also significantly reduced the formation of VDAC1 dimers without affecting VDAC1 expression (Figure 7I; Figure S16A, Supporting Information). These results indicated that Cd exposure increased the interaction between MCU and VDAC1 through MCU overexpression, which further promoted VDAC1 dimerization without changing the expression levels of VDAC1. Additionally, the functional inhibition of VDAC1 dimerization by VBIT4 obviously decreased LC3 II expression by ≈50% under Cd exposure, and inhibited the abundant formation of autophagosomes and autolysosomes, leading to improved hepatocyte viability (**Figure** **8**). In line with the effects of VBIT4, VDAC1 knockdown through siVDAC1 yielded similar results (Figures S2H and S17, Supporting Information). These results indicated that VDAC1 dimerization could intensively modulate Cd‐induced excessive mitophagy in its hepatotoxicity.

### 2.7 MCU Upregulation Induced Excessive Mitophagy by Promoting VDAC1 Ubiquitination

**Figure 9:**
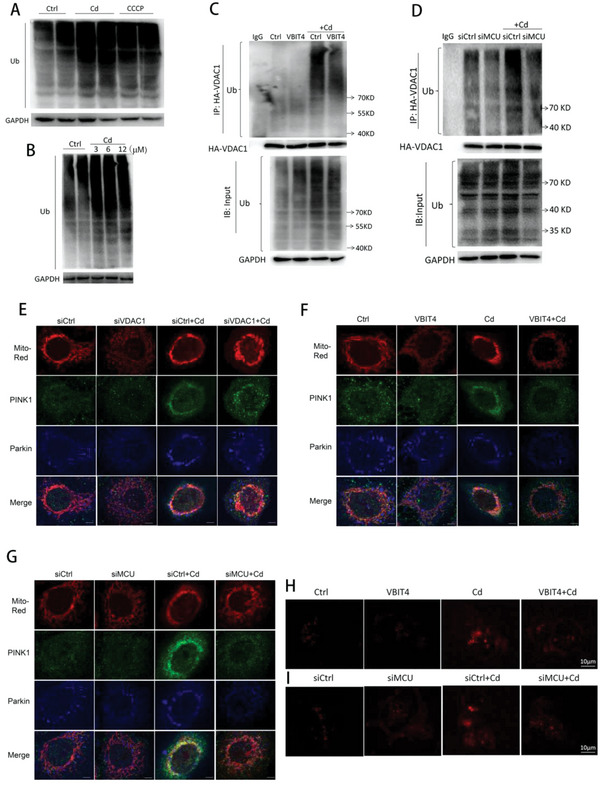
Mitophagy was inhibited by reducing VDAC1 ubiquitination through MCU downregulation. A) Immunoblots showing total ubiquitination in Cd (12 µm, 12 h)‐ or CCCP (10 µm, 12 h)‐treated cells. B) Immunoblots of total ubiquitination after 3, 6, and 12 µm Cd exposure for 12 h. C,D) Effects of VBIT4 (C) or siMCU (D) on the ubiquitination of total protein or VDAC1. Immunoprecipitation was performed using HA primary antibody to pull down HA‐VDAC1 with or without ubiquitin modifications. E–G) Confocal images indicating mitochondrial translocation of Parkin and PINK1 in cells pretreated with siVDAC1 (E), VBIT4 (F), and siMCU (G) before Cd exposure. Representative images are shown. The Parkin and PINK1 primary antibodies were mixed and incubated together before probing with two different secondary fluorescent antibodies. Scale bar: 5 µm. H,I) Representative confocal images indicating mitophagy after VBIT4 (H) or siMCU (I) treatment. Mitophagy dye was utilized to monitor the occurrence of mitophagy as described previously.

Finally, we deeply investigated the role of upregulated MCU in VDAC1‐involved mitophagy. Ubiquitination of VDAC1 induced by Parkin and PINK1 was responsible for the activation of mitophagy to clear dysfunctional mitochondria.^[^ ^19^ ^]^ Based on previous comprehensive data, we speculated that MCU upregulation accelerated VDAC1 ubiquitination‐mediated excessive mitophagy in Cd‐induced hepatotoxicity. Immunoblots revealed that Cd exposure enhanced total ubiquitination, similar to CCCP, a strong stimulator of ubiquitination (**Figure** **9**A), in a seemingly dose‐dependent manner (Figure 9B). Moreover, immunoprecipitation (IP) data revealed that Cd exposure also enhanced VDAC1 mono‐ or multi‐ubiquitination (Figure 9C,D). Genetic knockdown of Parkin effectively suppressed these (Figures S2K and S16B, Supporting Information). Interestingly, VBIT4, which inhibited VDAC1 dimerization, inhibited not only total ubiquitination but also VDAC1 ubiquitination (Figure 9C). Additionally, the knockdown of MCU by siMCU suppressed both total and VDAC1 ubiquitination induced by Cd (Figure 9D). We next mainly observed the expression and mitochondrial translocation of Parkin, PINK1, and p62, an adaptor that can recognize and attach to ubiquitinated VDAC1 and be further recognized by LC3 II before mitophagy initiation.^[^ ^20^ ^]^ As a result, Cd exposure increased PINK1 protein levels, decreased p62 protein levels, and promoted the translocation of Parkin, PINK1, and p62 into mitochondria (Figure S18A–C, Supporting Information), which was reversed by the application of siVDAC1, VBIT4 or siMCU (Figure 9E–G; Figure S19, Supporting Information). Additionally, VBIT4 and siMCU efficiently suppressed excessive mitophagy caused by Cd in HepG2 cells (Figure 9H,I), and the inhibitory effects of siMCU on excessive mitophagy in HepG2 cells were consistent with those results in primary liver cells with MCU knockout (Figure S8, Supporting Information). These collective data innovatively demonstrated that MCU upregulation promoted excessive mitophagy by increasing VDAC1 dimerization and then its ubiquitination in the hepatotoxicity of Cd.

## 3 Discussion

In this study, we revealed a novelty molecular mechanism highlighting that MCU triggered excessive mitophagy‐induced hepatotoxicity of Cd. After Cd exposure, the cytosolic Ca^2+^‐dependent increase and activation of CREB orchestrally upregulated MCU gene transcription and promoted translocation to mitochondria. The accumulation of MCU in mitochondria directly interacted with VDAC1 and further enhanced the dimerization and ubiquitination of VDAC1, which then overactivated mitophagy and lead to hepatocyte death (Figure S20, Supporting Information). Therefore, limiting MCU upregulation could be a novel approach to attenuate Cd‐induced hepatotoxicity.

Mitochondrial calcium uptake determines mitochondrial metabolism and cellular homeostasis in physiology and disease.^[^ ^6^ ^]^ Among channels exchanging Ca^2+^ between mitochondria and the rest of the cell, MCU serves as the essential channel for mitochondrial Ca^2+^ uptake. In recent years, the functional role of MCU in diverse diseases, such as cancerogenesis, heart failure, neurodegeneration, and muscle diseases, has been intensively investigated, and many inhibitors targeted to MCU have been developed for the treatment of these diseases.^[^ ^21^ ^]^ Regarding the abundant existence and importance of mitochondria in liver tissues, the close interaction between mitochondrial Ca^2+^ signaling and liver diseases has attracted increasing attention, implying the crucial role of MCU in these mitochondria‐associated liver diseases. In this study, we found that Cd exposure induced MCU upregulation and mitochondrial Ca^2+^ overload, and inhibition of MCU in vivo and vitro efficiently attenuated overactive mitophagy, inhibited mitochondrial and liver dysfunction, and improved cell viability and liver pathological conditions. Combined with other's studies, it demonstrated that the hepatotoxicity of Cd could be prevented or cured by targeting to MCU and mitochondrial calcium uptake.^[^ ^5^, ^22^ ^]^ Interestingly, in the neurotoxicity of Cd, Cd exposure was not able to increase MCU protein levels and to enhance mitochondrial calcium uptake, and MCU KO failed to attenuate neural death.^[^ ^22a^ ^]^ The discrepancy may be due to tissue‐specificity or context‐dependence. MCU complexes synergistically regulate MCU activity and avoid mitochondrial Ca^2+^ overload.^[^ ^23^ ^]^ In hepatotoxicity of Cd, we found that EMRE expression was upregulated while that of MCUb, MICU1, and MICU2 were not altered. The increased expression of EMRE would enhance MCU activity importing mitochondrial Ca^2+^. More importantly, the in vitro and in vivo inhibition of MCU efficiently attenuated autophagic cell death caused by Cd. These results demonstrated that MCU worked as the key molecule of MCU complexes in excessive mitophagy‐induced hepatotoxicity of Cd. Prospectively, our study paved the therapeutic avenue targeting MCU for the hepatotoxicity of Cd.

It is context dependent that whether MCU‐modulated mitophagy is beneficial or harmful. For instance, at basal levels, MCU mediated the initiation of mitophagy to remove damaged mitochondrial sectors to maintain mitochondrial and cellular homeostasis, which was beneficial.^[^ ^11^ ^]^ In many liver diseases caused by ethanol, acetaminophen, polyethylenimine or anorexia nervosa, mitophagy is activated and improves disease conditions by clearing out damaged mitochondria and maintaining cellular homeostasis.^[^ ^24^ ^]^ In these liver diseases, the injurious stresses tended to be chronic and less virulent. Conversely, our study revealed that upregulated MCU promoted excessive mitophagy‐mediated hepatotoxicity of Cd, thus presenting as detrimental. We speculated that Cd‐induced excessive and detrimental mitophagy was probably attributed to the high sensitivity of hepatocytes to Cd, the strong toxicity and the long‐term exposure duration of Cd. Consistent with our finding, I/R or LaCl similarly induced neurocyte death through upregulated MCU‐induced mitophagy overactivation because neurocytes were sensitive to I/R or LaCl treatment when exposed for a relatively long duration.^[^ ^12^ ^]^

Notably, our study delineated a novel molecular mechanism by which MCU upregulation controlled excessive mitophagy through VDAC1 in the hepatotoxicity of Cd. VDAC1 was the most abundant adaptor protein on the OMM that regulated mitochondrial metabolism by controlling transport of ions and small molecules through the OMM.^[^ ^16^ ^]^ Mitophagy could be initiated through VDAC1 ubiquitinated by PINK1 and Parkin, which translocate into the OMM through mitochondrial depolarization‐dependent biochemical signaling. In our study, VBIT4 inhibited the mitochondrial translocation of PINK1, Parkin, and p62, this implied that VBIT4 suppressed the mitochondrial depolarization (manifested as MMP reduction) caused by Cd. Thus we speculated that VBIT4 inhibited VDAC1 ubiquitination through reducing VDAC1 dimerization, decreasing mitochondrial ions intake and finally suppressing mitochondrial depolarization.

We here demonstrated that three classic mPTP components (CypD/ATPase/ANT) had no connection with MCU upregulation‐induced autophagic cell death in hepatotoxicity of Cd. It was different from that mPTP promoted Cd‐induced apoptosis and necrosis.^[^ ^25^ ^]^ Regarded as an essential component, VDAC1 regulated the opening of mPTP. However, the molecular nature of mPTP is contentious.^[^ ^26^ ^]^ Whether VDAC1 was necessary for mPTP was disputed. Instead, other important functions of VDAC1 have been uncovered in recent years rather than its role as an mPTP constituent.^[^ ^27^ ^]^ Under normal conditions, VDAC1 was widely distributed on the OMM in a form of monomer. Reversely, they were usually aggregated as oligomers in cancer, inflammatory and apoptosis‐related diseases.^[^ ^27a^ ^]^ The oligomers of VDAC1 significantly enhanced the transporting ability and further affected mitochondrial metabolism.^[^ ^27^ ^]^ In our study, Cd exposure mainly induced VDAC1 dimerization like that found in other experimental models with As_2_O_3_, H_2_O_2_, cisplatin or selenite treatment.^[^ ^18^ ^]^ It indicates that dimerization is a dominating, easy‐forming and more stable state compared to other VDAC1 aggregates (e.g., trimer, tetramer) in pathological conditions. In addition, after Cd exposure, VDAC1 formed dimers in the absence of VDAC1 upregulation, which was different from other findings that the dimerization of VDAC1 usually depended on its own upregulation in many pathological states such as NAFLD, cancer, Alzheimer's disease, and cardiovascular diseases.^[^ ^27^, ^28^ ^]^ Further, dimerization of VDAC1 promoted its ubiquitination based on the fact that a specific inhibitor of VDAC1 oligomerization VBIT4 successfully inhibited VDAC1 ubiquitination in Cd's hepatotoxicity.^[^ ^18^ ^]^

As far as the interaction between MCU and VDAC1, although previous studies implied the potential links between MCU and VDAC1, the significance for their interaction on mitophagy and the exact binding domains have never been explored.^[^ ^19^, ^29^ ^]^ Herein, we revealed that MCU directly interacted with VDAC1. In addition, our present study indicated that the binding sites between MCU and VDAC1 were not in IMS or TM domain of MCU. According to the previous study, MCU could bind to Miro1 (a protein on OMM) at N‐terminal domain (aa2‐57).^[^ ^30^ ^]^ MCU probably interacted with VDAC1 through its N‐ or C‐terminus. By disrupting their physical interaction at these domains would be an effective way to better explain MCU‐induced VDAC1 dimerization.

It is another significant issue to address how Cd upregulates MCU expression. CREB, as a general transcription factor, was proven to control MCU expression in different disease models, such as pathological cardiac remodeling, spinal morphine tolerance, and neuroblastoma cell death.^[^ ^31^ ^]^ The underlying regulatory mechanisms for CREB on MCU expression were still obscure. Herein, we reported that Cd exposure increased the protein levels of CREB and enhanced the phosphorylation of CREB^S133^. CREB was mainly localized at nucleus and was usually phosphorylated at Ser133 site by protein kinase activated by elevated cytoplasmic Ca^2+^.^[^ ^32^ ^]^ Once phosphorylated at Ser133 site, the transcriptional activity of CREB markedly increased and promoted the transcription of target genes. In addition, BATPA reversed the phosphorylation of CREB and the upregulation of MCU. This clarified that the regulatory effect of CREB on MCU was cytosolic Ca^2+^ dependent. Notably, we discovered three potential binding sequences, TGAGGTCT (‐1630‐1623), ACGTCA (‐599‐594), and CTCCGTGATGTA (‐495‐484), indicating that CREB could broadly modulate MCU gene transcription, and artificial manipulation of these three binding sequences to inhibit MCU‐induced excessive mitophagy seemed promising. The specific binding sites, such as the coexisting G/T base points among the three sequences, need further identification by site‐directed mutation.

## 4 Conclusion

Taken together, our study innovatively demonstrated that MCU upregulation by CREB promoted Cd‐induced hepatotoxicity by inducing excessive mitophagy through increasing VDAC1 dimerization and ubiquitination. The MCU is a potential therapeutic molecular target for the hepatotoxicity of Cd and other hepatic diseases involved in excessive mitophagy.

## 5 Experimental Section

### Animal Models

CD1 mice were used in this study. The method for breeding and genotyping *mcu* gene‐modified mice is summarized in Figure S6 of the Supporting Information. Briefly, *mcu* ^+/−^ (heterozygous, HZ) male and *mcu* ^+/‐^ female mice were mated, and the offspring, including *mcu* ^+/+^ (wild type, WT) and *mcu* ^+/‐^ were used for later experiments. Mice were housed in a specific pathogen‐free room with a 12‐h dark/light cycle at 23 ± 2 °C and fed standard mouse food ad libitum. Male mice aged 6–8 weeks were subjected to peritoneal exposure to saline or 5 mg kg^−1^ CdCl_2_ (202908, Sigma‐Aldrich, USA) once a day for 7 consecutive days. The exposure duration and Cd amount were chosen based on the previous studies and the pre‐experimental results from CD1 mice.^[^ ^4^, ^5^ ^]^ On the 8th day, mice were anesthetized and euthanized with chloral hydrate before whole blood and liver tissue harvest for histological and biochemistry analysis. All experiments were permitted by the Ethics Committee of the First Affiliated Hospital of Chongqing Medical and Pharmaceutical College.

### Cell Lines

The HepG2 cell line was purchased from the Cell Bank of the Institute of Biochemistry and Cell Biology (Shanghai, China). The HepG2 cells were cultured at 37 °C and 5% CO_2_ in MEM (10‐010‐CVR, Corning) containing 10% fetal bovine serum (FBS; FBSSA500‐S, AusgeneX) and 1% penicillin–streptomycin (SV30010, HyClone). HEK293T cells were grown at 37 °C and 5% CO_2_ with DMEM (10‐013‐CV, Corning) supplemented with 10% FBS and 1% penicillin–streptomycin. These cells were passaged every 2–3 days to maintain viability. To establish a stable VDAC1‐overexpressing HepG2 cell line, HepG2 cells were infected with the indicated lentivirus (GeneChem, Shanghai, China) containing the human VDAC1 gene fused with an HA tag (a small tag protein with a molecular weight of 1 KD) and purified by puromycin (2 ng mL^−1^; ST551, Beyotime, China). HA‐VDAC1 HepG2 cells were grown in the same conditions as HepG2 cells. The morphology of these cell lines was monitored to guarantee reliable results.

### siRNAs and Plasmid Transfection

Three siRNAs for MCU, CREB, CypD, VDAC1, ATG5, PINK1, ATP5G1, ANT2, and Parkin, including the negative control, were designed and synthesized by RIBO (Guangzhou, China). All plasmids or scramble vectors were constructed by GeneChem (Shanghai, China). Immunoblotting was used to confirm the knockdown effect of each siRNA or the upregulation effect of the indicated plasmid after transfection into cells by Lipofectamine 3000 (L3000075, Invitrogen) and Opti‐MEM (31985088, Gibco) for 48 h. Based on the immunoblotting results, the siRNA with the highest knockdown effect was used in subsequent experiments (Figure S3, Supporting Information). The target sequence of the most effective siRNA is shown in Table S3 of the Supporting Information. According to the study design, siRNA or plasmid could be previously transfected into cells for 24–48 h at different amounts.

### Mitophagy Monitoring

The Mitophagy Detection Kit (MD01, Dojindo) was chosen for mitophagy evaluation. Cells were washed twice with serum‐free MEM and stained with mitophagy dye (100 nm in serum‐free MEM) for 30 min at 37 °C and 5% CO_2_. The cells were washed twice again and exposed to CdCl_2_ for 12 h before the third wash and staining with lysosome dye (1 µm in serum‐free MEM) for 30 min at 37 °C and 5% CO_2_. The images were taken by confocal microscopy (SpinSR, Olympus) at 488 nm (green, indicating lysosomes) and 561 nm (red, indicating mitochondrion). The mitophagy intensity was related to the brightness of the red color overlapping with lysosomes. After mitochondrial staining, the cells were treated with Ru360 (5 µm; 557440, Sigma‐Aldrich, USA) for 2 h before CdCl_2_ exposure. However, cells were transfected with siMCU for 48 h before mitochondrial staining. Ru360 and siMCU were both utilized to assess the effect of MCU on mitophagy caused by CdCl_2_.

### Autophagic Flux

The autophagic double‐labeled adenovirus EGFP‐mCherry‐LC3 was obtained from HANBIO (Shanghai, China). According to the manufacturer's instructions, adenovirus titer, and cell number, HepG2 cells were properly infected at a multiplicity of infection of 50. After infection for 24–48 h and exposure to CdCl_2_ for 12 h, the cells were washed and fixed, followed by observation under confocal microscopy at 488 nm (green) and 561 nm (red). The separate or merged images were analyzed by counting the yellow dots representing autophagosomes and the free red dots representing autolysosomes in each cell (*n* = 15–20). The intensity of autophagic flux was positively related to yellow and/or red dot counts. To assess the effects of the indicated drugs or siRNAs on autophagic flux, prior to adenovirus infection and CdCl_2_ exposure, the cells were pretreated with the autophagy inhibitor 3‐methyladenine (3‐MA, 2 mm; HY‐19312, MCE), bafilomycin A1 (Baf A1, 50 nm; HY‐100558, MCE), Ru360 (5 µm), VBIT4 (10 µm; HY‐108937, MCE) or BAPTA‐AM (20 µm; HY‐100545, MCE) for 2 h and were transfected with siMCU, siCypD or siVDAC1 for 24 h.

### Immunoblotting

Cells or fresh liver tissues were lysed in cold RIPA buffer (P0013C, Beyotime) containing protease and phosphatase inhibitors (4693116001, 5892970001, Roche, respectively). The lysates were centrifuged at 12 000 × *g* at 4 °C for 15 min, and the protein concentration was determined by BCA assays before denaturation (95 °C, 10 min) and sodium dodecyl sulfate (SDS)–polyacrylamide gel electrophoresis (SDS–PAGE) loaded with 20–30 µg of sample. The proteins on gels were transferred to a polyvinylidene fluoride membrane (0.22 µm), blocked in blocking buffer (P0252, Beyotime), and incubated with the indicated primary antibodies overnight at 4 °C and the corresponding secondary antibodies for 1 h at room temperature before visualization by enhanced chemiluminescence (Millipore). The protein levels were indirectly quantified by ImageJ software. The primary antibodies were used as follows: GAPDH (1:5000; ab181602, Abcam), *β*‐tubulin (1:1000; 2148, CST), actin (1:1000; ab8226, Abcam), LC3B (1:1000; L7543, Sigma‐Aldrich), SQSTM1 (p62, 1:1000; 39749, CST), Parkin (1:1000; 4211, CST), PINK1 (1:1000; 23274‐1‐AP, Proteintech), ANT2 (1:500; 14671, CST), ATP5G1 (1:500; T57254S, Abmart), ATG5 (1:1000; 12994, CST), MCU (1:1000; 14997, CST), EMRE (1:500; ab122209, Abcam), MCUb (1:500; HPA048776, Sigma‐Aldrich), MICU1 (1:500; 12524, CST), MICU2 (1:500; HPA045511, Sigma‐Aldrich), CREB (1:1000; 9197, CST), p^s133^CREB (1:1000; 9198, CST), VDAC1 (1:500; 4661, CST), CypD (1:1000; ab110324, Abcam), HA (1:1000; 3724, CST), MYC (1:1000; 16286‐1‐AP, Proteintech), and ubiquitin (1:1000; 43124, CST).

### Coimmunoprecipitation

Owing to the relatively lower expression of VDAC1 compared with MCU in HepG2 cells (as confirmed by immunoblot), the HA‐VDAC1‐overexpressing HepG2 cell line was established to evaluate the interaction between MCU and VDAC1 by coimmunoprecipitation (Co‐IP). Cells were lysed in cold SDS‐free lysis buffer (P0013, Beyotime) containing protease and phosphatase as described above. The total proteins were separated by centrifugation at 12 000 × *g* and 4 °C for 15 min. The original protein concentration was detected by a BCA kit followed by partial denaturation. The MCU, HA or isotype IgG (5 µg) was incubated and fully mixed with 500 µL of 0.5% PBST (PBS with Tween 20) containing 50 µL of protein A/G magnetic beads (HY‐K0202, MCE) and 1 mm PMSF (ST506, Beyotime) for 3 h at 4 °C. After that, the magnetic beads were collected using a magnetic separator and further incubated and mixed with natural protein samples (0.5–1.0 mg) harvested previously in 500 µL of binding buffer for 12–18 h at 4 °C. The magnetic beads were collected again by magnetic separation and heated at 95 °C for 5 min, and the denatured proteins bound to the beads were harvested for immunoblots.

### VDAC1 Ubiquitination

VDAC1 ubiquitination was detected by IP. HA‐VDAC1‐overexpressing HepG2 cells were washed twice with precooled PBS, lysed in SDS‐free lysis buffer containing 20 µm N‐ethylmaleimide (NEM, HY‐D0843, MCE), and centrifuged at 12 000 × *g* for 15 min. Protein levels in the supernatants were detected as described above, and part of the protein sample was separated for determination of total ubiquitination. The remaining 500 µg or more protein samples in 1% SDS were denatured at 95 °C for 10 min, and the concentrations were determined after adjusting the SDS concentration to 0.1%. Equal quantities of protein samples were incubated with magnetic beads coated with HA primary antibody at 4 °C for 12–18 h for specific isolation and ubiquitination detection of VDAC1. The ubiquitin antibody (1:1000) was applied for total and VDAC1 ubiquitination detection, and the VDAC1 level in the IP product was also confirmed by immunoblotting.

### MCU Quantitative Polymerase Chain Reaction

Total RNA was isolated using TRIzol and stored at −80 °C until use. An Omniscript RT kit (205110, QIAGEN) was used for cDNA synthesis. Briefly, the template RNA (50 ng to 2 µg) was added to master mix containing buffer, dNTP mix, specific primers, RNAase inhibitor, and Omniscript reverse transcriptase and incubated for 60 min at 37 °C after mixing thoroughly. Less than 5 µL of the finished reverse‐transcription reaction containing cDNA was used for quantitative polymerase chain reaction (qPCR) by a QuantiNova SYBR green PCR kit (208052, QIAGEN) in a real‐time cycler (Bio‐Rad). The primer sequences are provided in Table S3 of the Supporting Information. MCU mRNA expression values were normalized to GAPDH, and the relative expression level was computed using standard methods (2^−ΔΔCt^) with three independent technical repeats.

### Immunofluorescence

The cells seeded on cover glasses in 6‐well plates were treated with or without 200 nm MitoTracker Red CMXRos (M7512, Thermo Fisher Scientific) for 30 min at 37 °C and 5% CO_2_. Then, the cells were fixed at room temperature for 10 min, blocked in blocking buffer (P0260, Beyotime) at room temperature for 20 min, incubated with the indicated primary antibodies (MCU, 1:300; p^s133^CREB, 1:500; HA, 1:300; MYC, 1:300; Parkin, 1:250; p62, 1:250; PINK1, 1:250) overnight at 4 °C, and incubated with secondary antibodies (Goat anti‐rabbit 488, 150077, Abcam; Goat anti‐mouse 405, 175660, Abcam) at room temperature for 1 h. A mixture of two different primary or secondary antibodies was prepared for double staining of two different proteins, Parkin and p62. Cells on coverslips were placed on slides after the addition of sealing liquid to avoid fluorescence quenching. DAPI (10 µg mL^−1^) was used for nuclear labeling at room temperature for 10 min. Images were obtained under a confocal microscope at 63× magnification according to the properties of the secondary antibodies.

### Detection of Mitochondrial Calcium by GCaMP6m Plasmids

The HepG2 cells in confocal dishes were transfected with GCaMP6m constructs for 24 h before treatment with or without 12 µm Cd for 12 h. As to increased MCU expression was positively correlated with mitochondrial fission, these cells were thus selected to assess alterations of mitochondrial calcium in Cd‐treated group. The fluorescence was consecutively observed and recorded at 475 nm (ex) and 410 nm (ex) under the confocal microscopy according to the methods previously reported.^[^ ^33^ ^]^ During observation, histamine (100 µm; HY‐B1204, MCE) was added into dishes to stimulate accumulation of mitochondrial calcium. The 475/410 ratio (ratio of average gray value) was computed for further analysis.

### Apoptosis and Necrosis Analysis

This experiment was performed by Apoptosis and Necrosis Assay Kit (C1056, Beyotime) according to the manufacturer's instructions. Briefly, HepG2 cells were seeded onto glass slides overnight and were subjected to indicate treatments. The cells were then washed once and stained with hoechst 33342 and propidium iodide (PI) for 20 min at 4 °C, and the blue and red fluorescence signals were observed under inverted fluorescence microscope. For each group, 6 random fields (100×) were collected for statistical analysis. Normal cells showed weak blue and red signal, apoptotic cells showed strong blue and weak red signal, and necrotic cells showed strong blue and red signal.

### TMRM Stain

It was reported that TMRM was an indicator for mitochondrial membrane potential and could reflect the alteration of MPTP. As a positive control, ionomycin (10 µm; HY‐13434, MCE) was incubated with cells pretreated with indicated siRNAs or inhibitors for 30 min at 37 °C. Next, treated cells were stained by TMRM (1 µm; HY‐D0984, MCE) for 30 min at 37 °C followed by washing once in culture medium. The fluorescence intensity was detected at 550/575 nm (ex/em) on a microplate reader. The CypD inhibitor CSA (10 µm; HY‐B0579, MCE), ATP synthase inhibitor oligomycin (10 µm; SC0366, Beyotime), and ANT2 inhibitor bongkreic acid (10 µm; SC‐205606, Santa Cruz) were used in this assay.

### mPTP Opening Assay

The mPTP Assay Kit (C2009S, Beyotime) was used to assess the opening of mPTP opening. This was a classical and efficient method widely used by researchers. Briefly, Calcein AM (calcein acetoxymethyl ester) was preloaded with HepG2 cells treated with indicated siRNAs in black 96 well plates. After that, cells were incubated with CoCl_2_ and ionomycin at 37 °C for 30 min to quench the fluorescence in cytoplasm. Ionomycin was utilized as a positive control that opened mPTP. The fluorescence intensity was finally detected at 494/517 nm (ex/em) on a microplate reader.

### Isolation of Mouse Primary Liver Cells

Primary liver cells from WT and KO CD1 mice were isolated by kit (LV Biotech, China) according to the manufacture's protocol with some modifications. Briefly, mice were anesthetized by intraperitoneal injection of 4% chloral hydrate and further sterilized by 75% ethanol. In a clean bench, the mouse liver, postcava, and portal vein were fully exposed. Soon after clamping the precava and then cutting of the portal vein, the preheated EGTA and collagenase solution was consecutively pumped into liver via the postcava within 7 min for each. Then the digested liver was carefully removed and further digested in the collagenase solution in a 10 cm dish for 2 min. The liver cell suspensions were filtrated through a 70 mesh cell strainer after adding 20 mL cold washing buffer. Finally, the primary liver cells were collected by centrifugation and resuspended in 15 mL DMEM cell culture for later experiments.

### ChIP

ChIP assays were performed by ChIP Assay Kit (P2078, Beyotime) according to the manufacturer's instructions. In brief, HepG2 cells in 10 cm dishes treated with or without Cd were treated with 1% formaldehyde at 37 °C for 10 min to crosslink proteins and DNA, followed with lysis in SDS buffer and ultrasonic shear of DNA. Next, 1 µg pCREB^S133^ primary antibodies were used to precipitate specific protein–DNA complexes followed with DNA purification and the final real‐time PCR assays. The PCR products were further analyzed on 1.2% agarose gel. The rabbit IgG was used as negative control. Values for each group were normalized by input DNA, and the enrichment of CREB on MCU promoter after Cd exposure was normalized by control group.

### FRET

The ECFP and the EYFP were selected as the donor and acceptor in FRET assay, respectively. The VDAC1‐ECFP and MCU‐EYFP plasmids were constructed by GeneCloudBiotech (Guangzhou, China) and cotransfected into HepG2 cells for 48 h. The fluorescence of ECFP and EYFP was observed and the latter was replaced with red before photobleaching of EYFP by 100% power at 514 nm (ex) over the course of 9 frames. Five regions of interest for FRET were analyzed in each group under a confocal microscopy (Leica, TCS SP5), and the average FRET efficiency were normalized to the ECFP+MCU‐EYFP group.

### Cell Viability

Cells were seeded on 96‐well plates and treated for the indicated periods before exposure to CdCl_2_. CCK‐8 reagent (CK04, Dojindo) was added to each well (10%) and incubated at 37 °C for 1 h. The optical density value was determined by a microplate reader at 450 nm.

### Biochemistry

After treatment, the mice were deeply anesthetized by chloral hydrate to yield enough whole blood. Approximately 1–1.5 mL of blood was centrifuged at 3000 rpm for 10 min at room temperature to separate serum for glutamic‐pyruvic transaminase (ALT), glutamic oxalacetic transaminase (AST), and lactic dehydrogenase (LDH) tests on a blood biochemistry analyzer (Beckman, AU5800).

### Liver Histology

The mice were consecutively perfused with precooled saline and 4% paraformaldehyde through the heart, and the fresh liver tissue was cut down and fixed with 4% paraformaldehyde for 72 h. Then, the tissues were demineralized and dehydrated, followed by paraffin embedding and sectioning into 5‐µm‐thick slices. The sections were stained with hematoxylin and eosin and observed under an inverted microscope (Olympus, Japan) to examine the pathological changes. For immunohistochemistry analysis of MCU expression in liver tissues, the dewaxed and hydrated liver sections were boiled for 20 min in 0.01 m sodium citrate buffer for antigen recovery. The H_2_O_2_ (3%) solution was used to inactivate peroxidase at room temperature for 10 min before the section was blocked with goat serum and successively incubated with MCU primary antibody (1:300) overnight at 4 °C and the HRP‐labeled secondary antibody for 1 h at room temperature. DAB substrates were used for visualization, and the nucleus was stained with hematoxylin before imaging.

### Transmission Electron Microscopy

Cells treated with or without CdCl_2_ were collected in Eppendorf tubes, washed twice in PBS, and fixed in 2.5% glutaraldehyde for 24 h. Liver tissues were quickly cut into small pieces of ≈2 × 2 mm^3^ at 4 °C, fixed in 2.5% glutaraldehyde for 24 h, and sectioned into ultrathin slices suitable for observation. Each 400 mesh copper screen was loaded with 3–4 slices for each sample. Samples were examined using a transmission electron microscope (Hitachi‐7500, Hitachi Company, Japan) at 20kx, and images were taken under a camera.

### Mitochondrial Isolation

Mitochondria were harvested using a mitochondrial isolation kit (89874, Thermo Fisher Scientific). Approximately 2 × 10^7^ cells were pelleted by centrifugation at 850 × *g* for 2 min. The pellet in a 2 mL microcentrifuge tube was moderately vortexed for 5 s after the addition of Reagent A (containing 1 mm PMSF) and incubated on ice for ≈2 min. Reagent B was then added, and the tube was sharply vortexed for 5 s. The suspension was incubated on ice for 5 min with a sharp vortex every minute. Then, Reagent C (containing 1 mm PMSF) was added to the tube, inverted and centrifuged for 10 min at 700 × *g* and 4 °C. The supernatant was transferred into another new tube followed by centrifugation for 15 min at 3000 × *g* and 4 °C. The supernatant was used for cytosolic content analysis. The remainder was suspended with Reagent C and centrifuged at 12 000 × *g* for 5 min at 4 °C, and the purified intact mitochondria were collected after discarding the supernatant. The mitochondrial pellet on ice was lysed with RIPA buffer for subsequent immunoblots.

### Mitochondrial Membrane Potential

Cells were washed twice with PBS and incubated with 10 µg mL^−1^ JC‐1 (C2005, Beyotime) in serum‐free culture medium for 20 min at 37 °C and 5% CO_2_. After incubation and washing, normal culture medium was added, and the fluorescence was observed under a microreader plate. The formation of monomers (green) and aggregates (red) was examined at 514/529 nm (excitation/emission wavelength) and 585/590 nm, respectively. The ratio between aggregates and monomer RFU was computed, which was positively related to the change in mitochondrial membrane potential.

### Mitochondrial ROS

Cells were washed once with PBS and incubated with 250 nm MitoTracker Red CM‐H2XRos (M7513, Invitrogen) dissolved in serum‐free culture medium for 30 min at 37 °C and 5% CO_2_. Serum‐free medium was added after washing, and the fluorescence (red) was immediately observed under a microreader plate at 579/599 nm. The production of mitochondrial ROS was positively related to the brightness of the red color.

### Intercellular ATP

The ATP determination kit (A22066, Invitrogen) was used for ATP detection. A standard reaction solution was prepared by the addition of dH_2_O, reaction buffer, and 0.1 m dithiothreitol, 10 mm d‐luciferin, and 5 mg mL^−1^ firefly luciferase based on the manufacturer's instructions. The reaction solution was well mixed and stored at 4 °C in the dark until use. A series of ATP concentrations ranging from 1 nm to 1 µm were utilized to produce a standard curve by adding 10 µL of ATP solution into 90 µL of standard reaction solution. The cell lysates in lysis buffer were prepared as described previously, and 10 µL of the lysate was added to 90 µL of standard reaction solution. The chemiluminescence value was obtained on a microreader plate. The ATP amount in the experimental sample was calculated from the standard curve.

### Dual‐Luciferase Reporter Assay

HEK293T cells (8000 per well) were seeded on 96‐well white plates and transfected with 50 ng of the indicated firefly luciferase reporter gene plasmid or (and) 50 ng of transcription factor (CREB or CREB^S133A^) plasmid, while the renal luciferase plasmid (5 ng per well) was also transfected as an internal control. After cotransfection for 48 h, the plates were placed at room temperature for 10 min, and 100 µL of firefly or Renilla luciferase substrate (RG088S, Beyotime) was added and incubated for 5 min each. The RLU, reflecting chemiluminiscence, were determined by a microplate reader, and the ratio of firefly to Renilla RLU was calculated for comparison between groups.

### VDAC1 Oligomerization

The HA‐VDAC1‐upregulated cells were washed with precooled PBS (pH 8.4), collected together in PBS (pH 8.4) with 300 µm [ethylene glycolbis (succinimidylsuccinate)] (EGS, 21565, Thermo Fisher Scientific), and incubated at 30 °C for 20 min for intercellular VDAC1 crosslinking. The crosslink reaction was quenched by the addition of 20 mm Tris (pH 7.5), and the cells were lysed for subsequent immunoblotting of VDAC1 oligomerization using HA antibody.

### Quantification and Statistical Analysis

Unless otherwise indicated, all continuous variables are expressed as the mean ± SD (standard deviation of variables) analyzed by SPSS 18.0. The immunoblot bands were analyzed using ImageJ and normalized to the control group. The dots were artificially counted for autophagic flux evaluation. Two‐sided Student's *t*‐test or analysis of variance (ANOVA) with post hoc tests was used to calculate the statistical significance between indicated groups. The chi‐square test was used for percentage comparison. Differences were considered statistically significant if *p* < 0.05 (*p < 0.05, **p < 0.01, ***p < 0.001).

## Conflict of Interest

The authors declare no conflict of interest.

## Author Contributions

S.C.X. and C.L. designed the project. C.L., H.J.L., W.X.D., Y.D., Q.Y., T.Z., Y.P.S., and Y.Y.L. performed the experiments and S.C.X., C.L., and H.J.L. analyzed the data. C.L. wrote the manuscript.

## Supporting information

Supporting Information

## Data Availability

The data that support the findings of this study are available from the corresponding author upon reasonable request.
