# Supplementary material for: MCU Upregulation Overactivates Mitophagy by Promoting VDAC1 Dimerization and Ubiquitination in the Hepatotoxicity of Cadmium
Source: Adv Sci (Weinh). 2023 Jan 15;10(7):2203869. doi: 10.1002/advs.202203869 (PMC9982555; doi:10.1002/advs.202203869)
Supplement: Supplementary file 1 — Supporting Information [file ADVS-10-2203869-s001.pdf]

ADVANCED SCIENCE
Open Access

Supporting Information

for *Adv. Sci.*, DOI 10.1002/advs.202203869

MCU Upregulation Overactivates Mitophagy by Promoting VDAC1 Dimerization and Ubiquitination in the Hepatotoxicity of Cadmium

*Cong Liu, Hui-Juan Li, Wei-Xia Duan, Yu Duan, Qin Yu, Tian Zhang, Ya-Pei Sun, Yuan-Yuan Li, Yong-Sheng Liu and Shang-Cheng Xu**

Supplementary Information for

MCU upregulation overactivates mitophagy by promoting VDAC1 dimerization and ubiquitination in the hepatotoxicity of cadmium

Cong Liu, Huijuan Li, Weixia Duan, Yu Duan, Qin Yu, Tian Zhang, Yapei Sun, Yuanyuan Li, Yongsheng Liu, Shangcheng Xu*

This file includes:

Figures S1 to S20
Tables S1 to S5

Figure S1

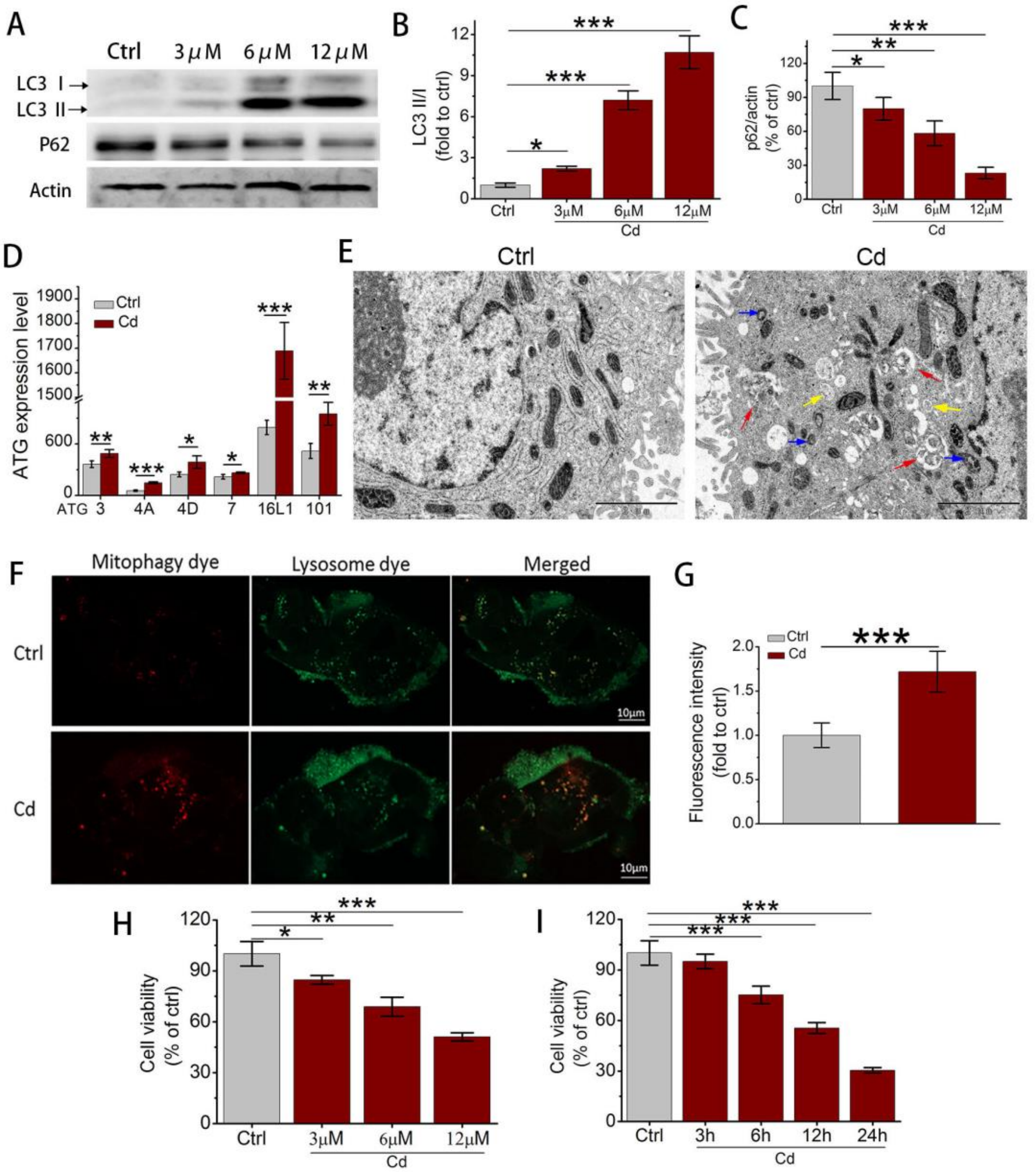

Supplementary figure 1: Upregulated mitophagy levels in Cd-induced hepatotoxicity. A) Immunoblots of LC3 Ⅱ/Ⅰ and p62. HepG2 cells were exposed to 3, 6 and 12 µM Cd for 12 hours, and whole-cell lysates were prepared for immunoblotting. B) The LC3 Ⅱ/Ⅰ ratio and C) The p62/ACTB ratio were normalized to the control group (*n* = 3). D) RNA-sequencing for ATGs. Cells were treated with or without 12 µM Cd for 12 hours. Total RNA was harvested for RNA-seq, and the alterations with statistical significance are shown (*n* = 4). E) TEM images of cells exposed to Cd or not. The blue arrow indicates an impaired mitochondrion, the yellow arrow indicates an autophagosome, and the red arrow indicates an autolysosome.

Scale bar, 2 μm. F) Confocal images of mitophagy caused by Cd exposure. Mitochondria and lysosomes were stained with the indicated dye. Scale bar, 10 μm. G) Fluorescence intensity of mitophagy dye (n = 10). H) HepG2 cells were exposed to 3, 6 and 12 μM Cd for 12 hours, and the cell viability was evaluated by CCK-8 assay (n = 5). I) Cells were exposed to 12 μM Cd for 3, 6, 12 and 24 hours respectively, followed by CCK-8 analysis (n = 5). *$p < 0.05$, **$p < 0.01$, ***$p < 0.001$.

Figure S2

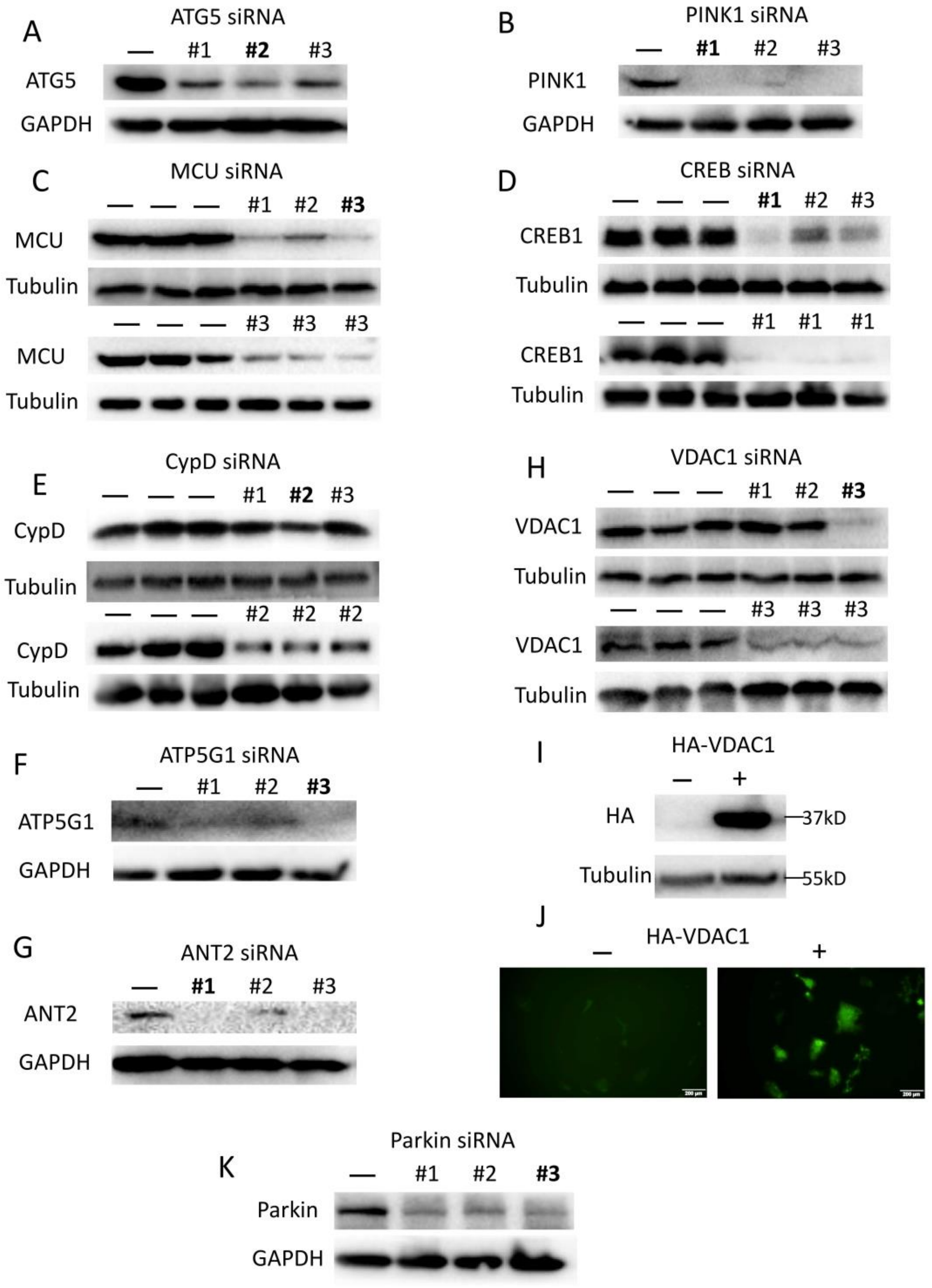

Supplementary figure 2: **Knockdown effect of siRNAs for ATG5, PINK1, MCU, CREB, CypD, ATP5G1, ANT2, VDAC1, Parkin and the identification of HA-VDAC1-overexpressed HepG2 cell line.** A to H) and K) Immunoblots for ATG5, PINK1, MCU, CREB, CypD, ATP5G1, ANT2, VDAC1 and Parkin in lysates from cells transfected with

negative control (NC) siRNA and three different designed siRNAs for 48 hours, respectively. The bold font indicated the most effective siRNA that was used in this study. As to MCU, CREB, CypD and VDAC1, we further verified the knockdown effect of selected siRNA with three replicates. I) Immunoblots for HA-VDAC1 in lysates from cells infected with lentivirus containing exogenous VDAC1 gene fused with HA-tag. The molecular weight was shown as indicated. J) Immunofluorescence staining of HA-VDAC1 in cells infected with HA-VDAC1 lentivirus or not, and the images were taken under inverted fluorescence microscope. Scale bar, 200 μm. Indicated primary and secondary antibodies were used in these immunoblots.

Figure S3

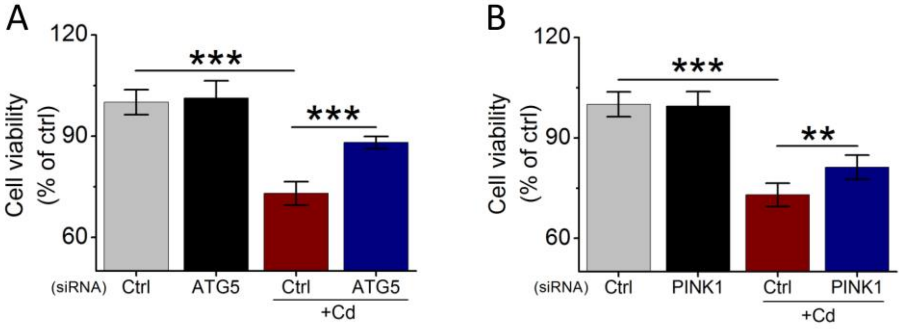

Supplementary figure 3: **ATG5 and PINK1 deficiency rescued Cd-induced cell death.** A) Cell viability after ATG5 depletion in Cd-treated HepG2 cells (*n* = 5). B) Cell viability after PINK1 knockdown in Cd-treated HepG2 cells (*n* = 5). **$p < 0.01$, ***$p < 0.001$.

Figure S4

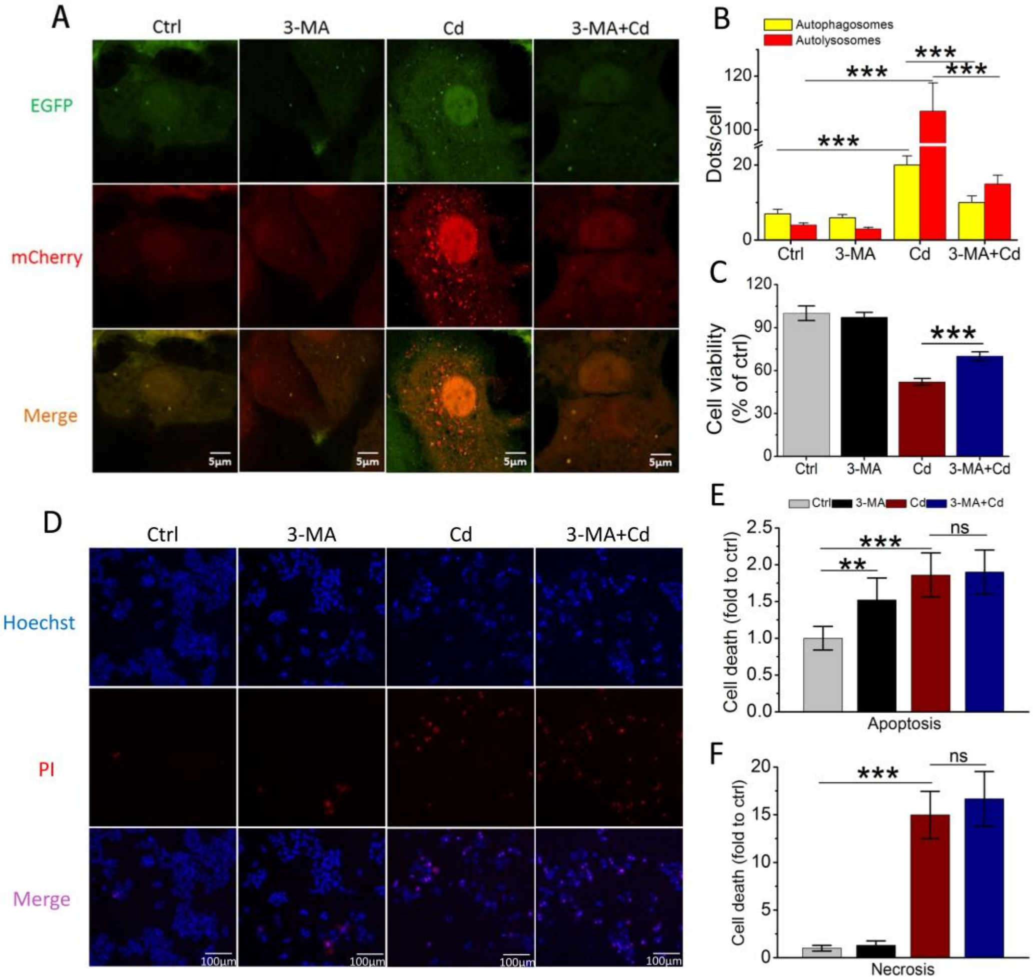

Supplementary figure 4: Effects of 3-MA on Cd-induced hepatotoxicity. A) Confocal images taken from HepG2 cells transfected with EGFP-mCherry-LC3 adenovirus 24 hours before 3-MA pretreatment and Cd exposure. Yellow dots indicate autophagosomes, and free red dots indicate autolysosomes. Scale bar, 5 µm. B) Quantification of autophagosomes and autolysosomes (n = 20). C) Cell viability after 3-MA treatment in Cd-exposed cells (n = 5). D) Analysis of apoptosis and necrosis in HepG2 cells by Hoechst 33342 (blue) and propidium iodide (PI, red) stain. 100 × magnification images were taken and representative results were shown. Scale bar, 100 µm. E) Apoptosis rate and F) necrosis rate were calculated from six 100 × magnification images. Normal cells showed as weak blue and red signal. Apoptotic cells showed as bright blue and weak red signal. Necrotic cells showed as bright blue and red signals. **$p < 0.01$, ***$p < 0.001$. ns, no significance.

Figure S5

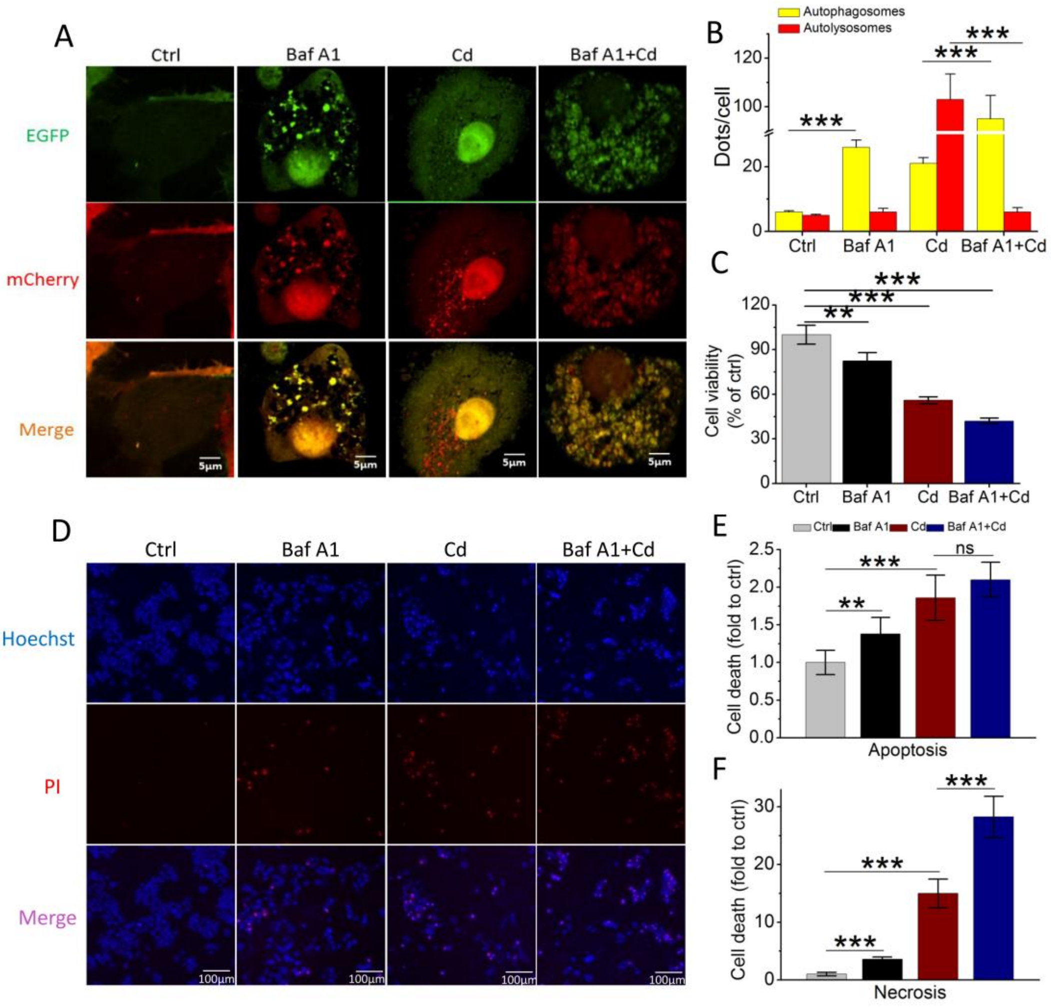

Supplementary figure 5: Effects of Baf A1 on Cd-induced hepatotoxicity. A) Confocal images taken from HepG2 cells transfected with EGFP-mCherry-LC3 adenovirus 24 hours before Baf A1 pretreatment and Cd exposure. Yellow dot indicated the autophagosome, and free red dot indicated the autolysosome. Scale bar, 5 μm. B) Quantification of autophagosomes and autolysosomes from (A), n = 20. C) Cell viability after Baf A1 treatment in Cd-treated cells (n = 5). D to F) Apoptosis and necrosis analysis as described above in S2 after Baf A1 treatment. $^{**}p < 0.01$, $^{***}p < 0.001$. ns, no significance.

Figure S6

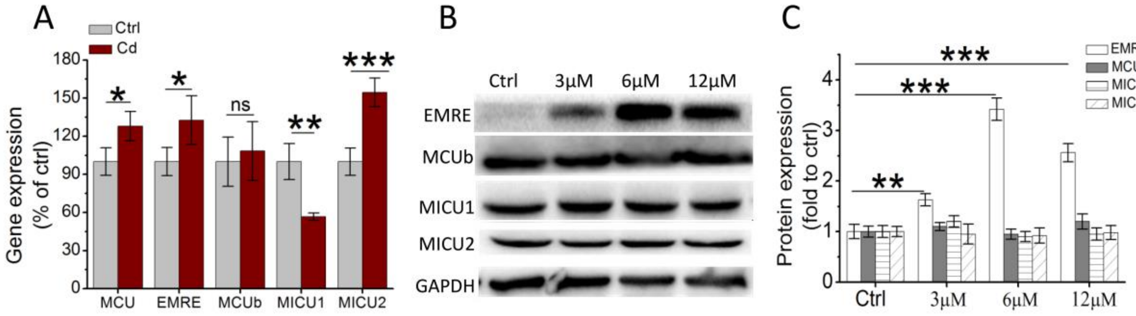

Supplementary figure 6: Gene and protein expression levels for MCU subunits. A) Gene expression levels of MCU, EMRE, MCUb, MICU1 and MICU2 after Cd exposure. The cells were exposed with or without 12 μM Cd for 12 hours, and the total RNA were harvested according to the requirments of RNA-seq (n = 4). B) Immunoblots for EMRE, MCUb, MICU1 and MICU2 in cells treated with or without 3, 6 and 12 μM Cd for 12 hours. GAPDH was used as the loading control. C) Quantitative analysis of (B). *$p < 0.05$, **$p < 0.01$, ***$p < 0.001$. ns, no significance.

Figure S7

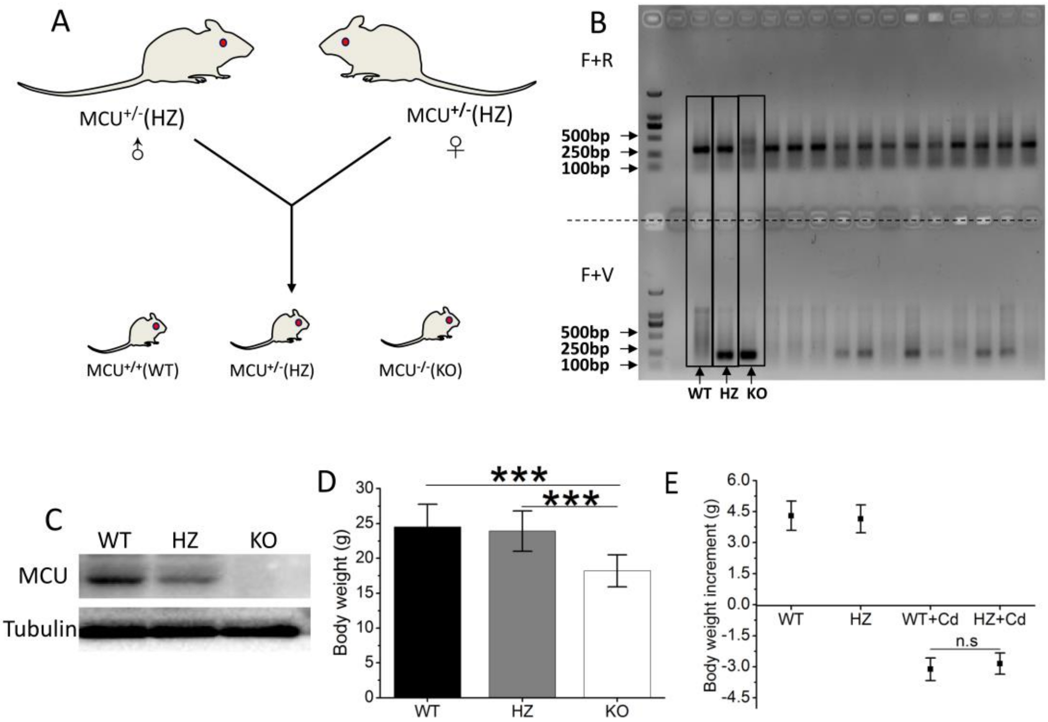

Supplementary figure 7: Breeding and genotyping of mcu gene modified CD1 mice. A) Schematic diagram for breeding of CD1 mice. Heterozygous (HZ) mice produced three genotypes, the wild type (WT) with patrilineal and maternal *mcu* gene expression, the heterozygous type with patrilineal or maternal *mcu* gene expression, and the knock out (KO) type without any *mcu* gene expression. B) Example of genotyping of WT, HZ and KO mice. Two pairs of primers, FR and FV, were designed for *mcu* genotyping. Mice tail was cut for harvest of DNA, followed by PCR and agarose gel electrophoresis. The WT, HZ or KO genotype was confirmed by existence of single FR signal (~300bp), both FR and FV signal (~200bp), and single FV signal, respectively. C) Immunoblots for MCU in liver lysates from WT, HZ and KO mice. D) Body weight of WT, HZ and KO mice at 6th week in normal conditions (n = 10). E) Body weight increment of WT or HZ mice treated with saline or Cd (n = 10). ***p < 0.001. ns, no significance.

Figure S8

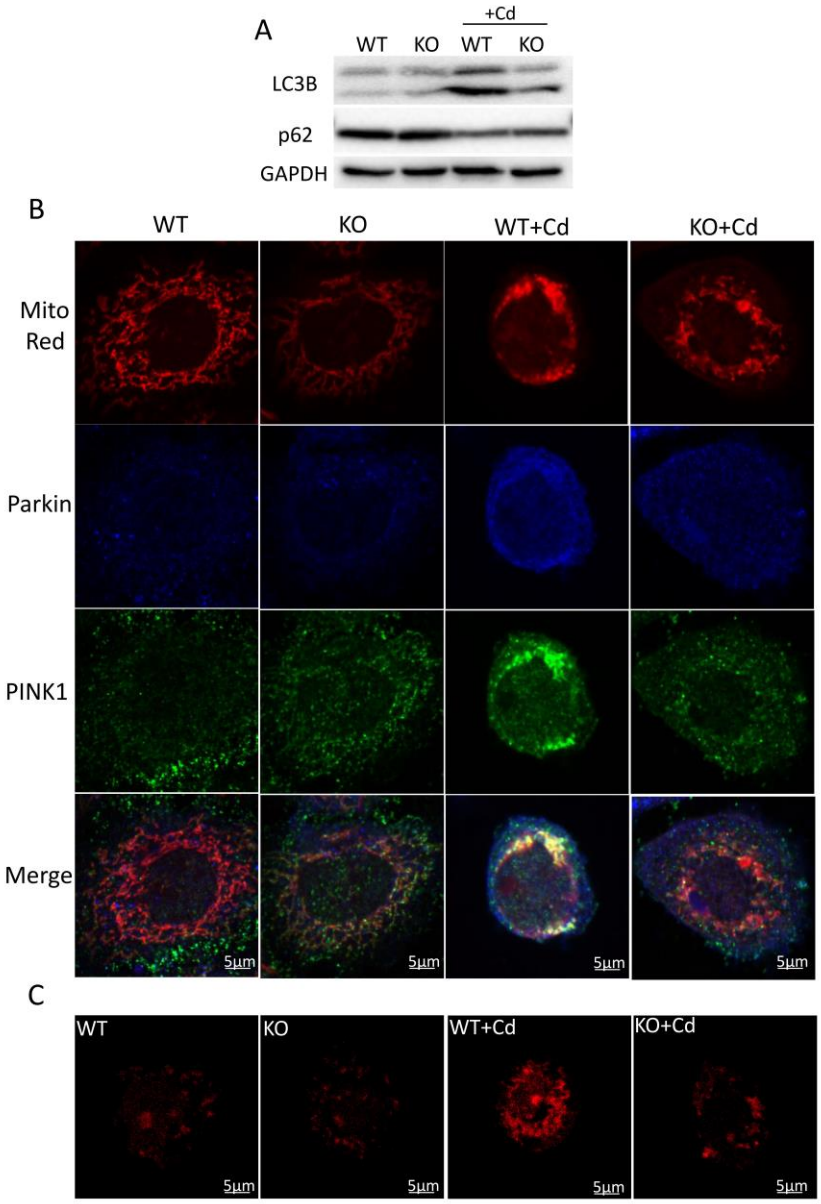

Supplementary figure 8: MCU knockout inhibited mitophagy caused by Cd in primary liver cells. The primary liver cells were isolated from the CD1 mice (WT and KO), and were subjected to Cd exposure for 12 hours within 3 days. A) Immunoblots of LC3 and p62 in cell lysates treated with or without Cd. B) Confocal images indicating the mitochondrial translocation of Parkin and PINK1. The Parkin and PINK1 primary antibodies were mixed and incubated together. C) Confocal images indicating the occurrence of mitophagy assessed by Mitophagy Dye. Representative results were given.

Figure S9

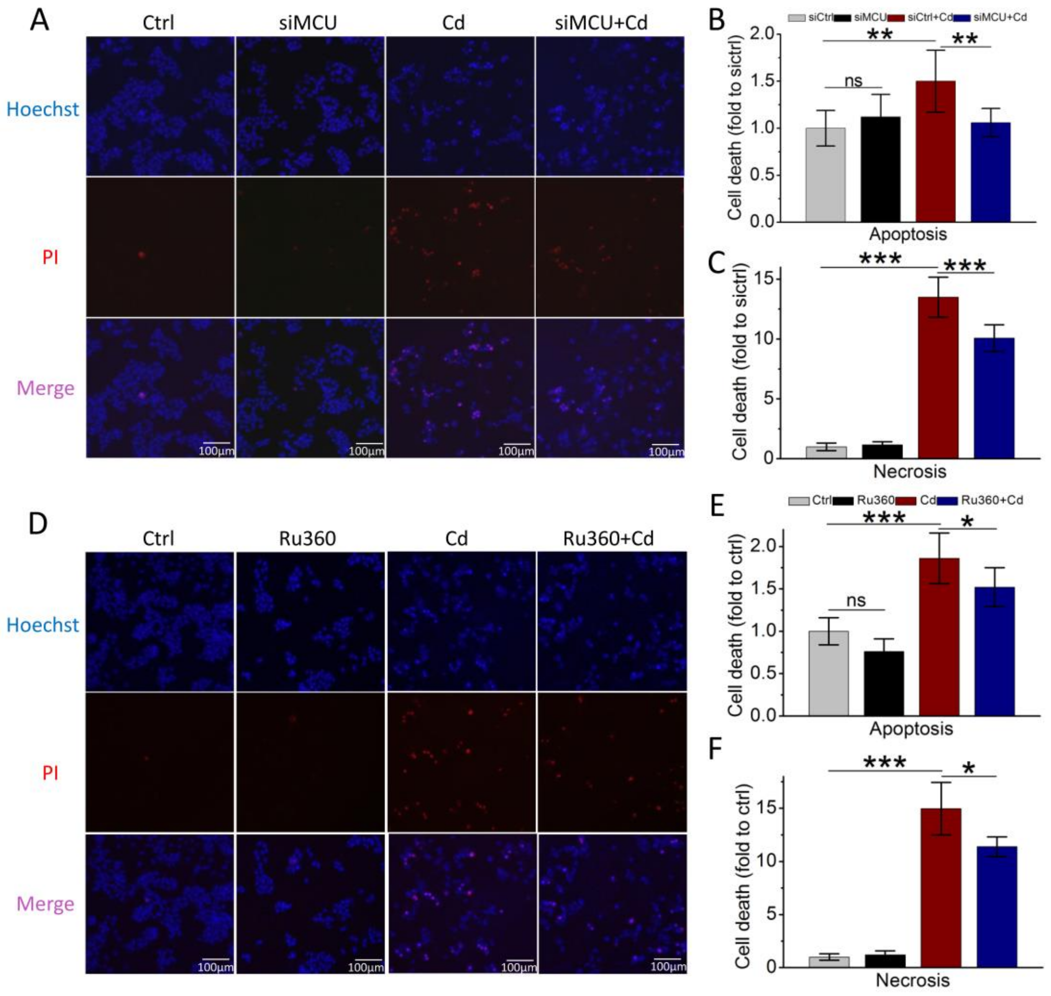

Supplementary figure 9: Effects of siMCU and Ru360 on Cd-induced apoptotic and necrotic liver cell death. A, D) Fluorescent images shown apoptotic and necrotic cell death after siMCU and Ru360 treatment, respectively. B, E) Apoptotic rate was calculated from six 100 × magnification images after siMCU and Ru360 treatment, respectively. C, F) Necrotic rate was also calculated from six 100 × magnification images after siMCU and Ru360 treatment, respectively. **p* < 0.05, ***p* < 0.01, ****p* < 0.001. ns, no significance.

Figure S10

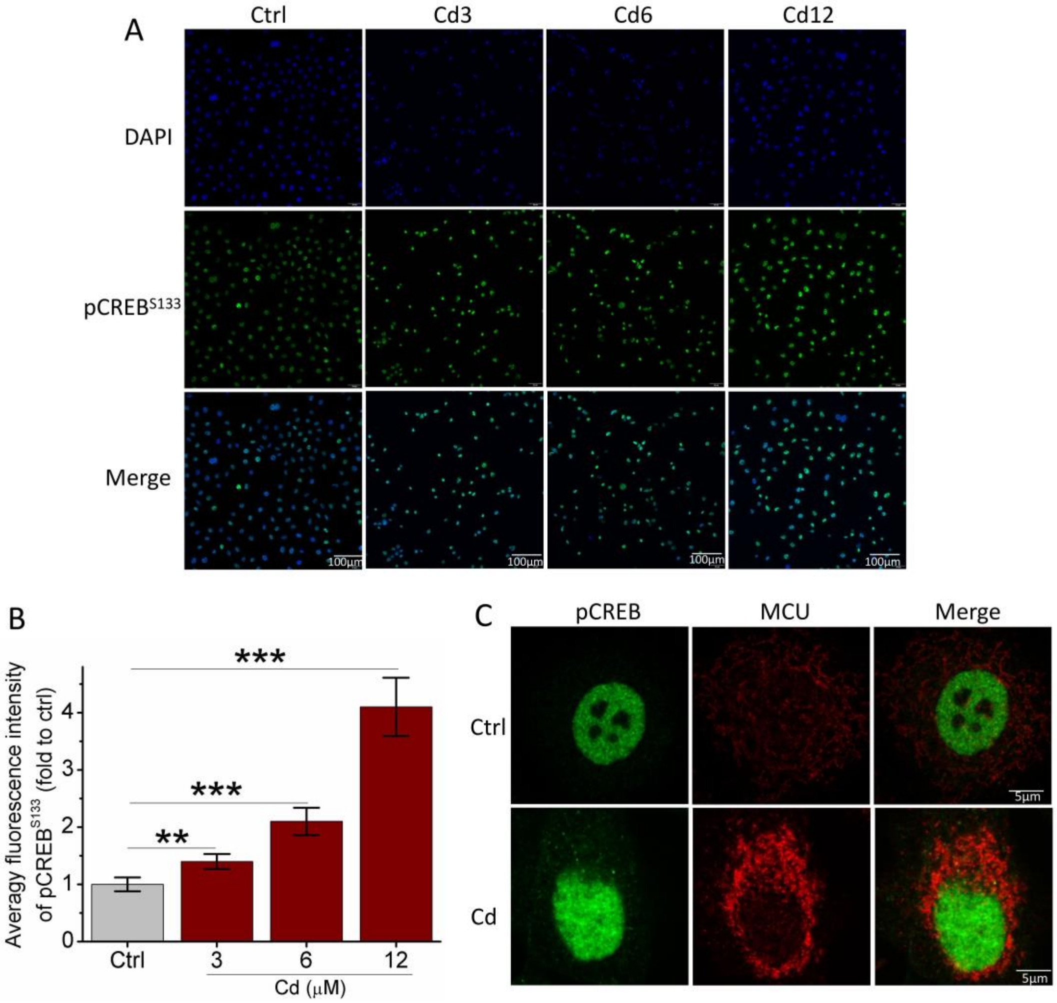

Supplementary figure 10: Nuclear translocation of pCREB^{S133} promoted MCU expression. A) Confocal images indicating nuclear translocation of pCREB^{S133} after 3, 6 and 12 μM Cd treatment in HepG2 cells. Nucleus was stained with DAPI. B) Average fluorescence intensity of nuclear pCREB^{S133} in each cell treated with different dose of Cd (n = 20). C) Confocal images reflecting nuclear translocation of pCREB^{S133} and MCU expression after 12 μM Cd exposure for 12 hours. **$p < 0.01$, ***$p < 0.001$.

Figure S11

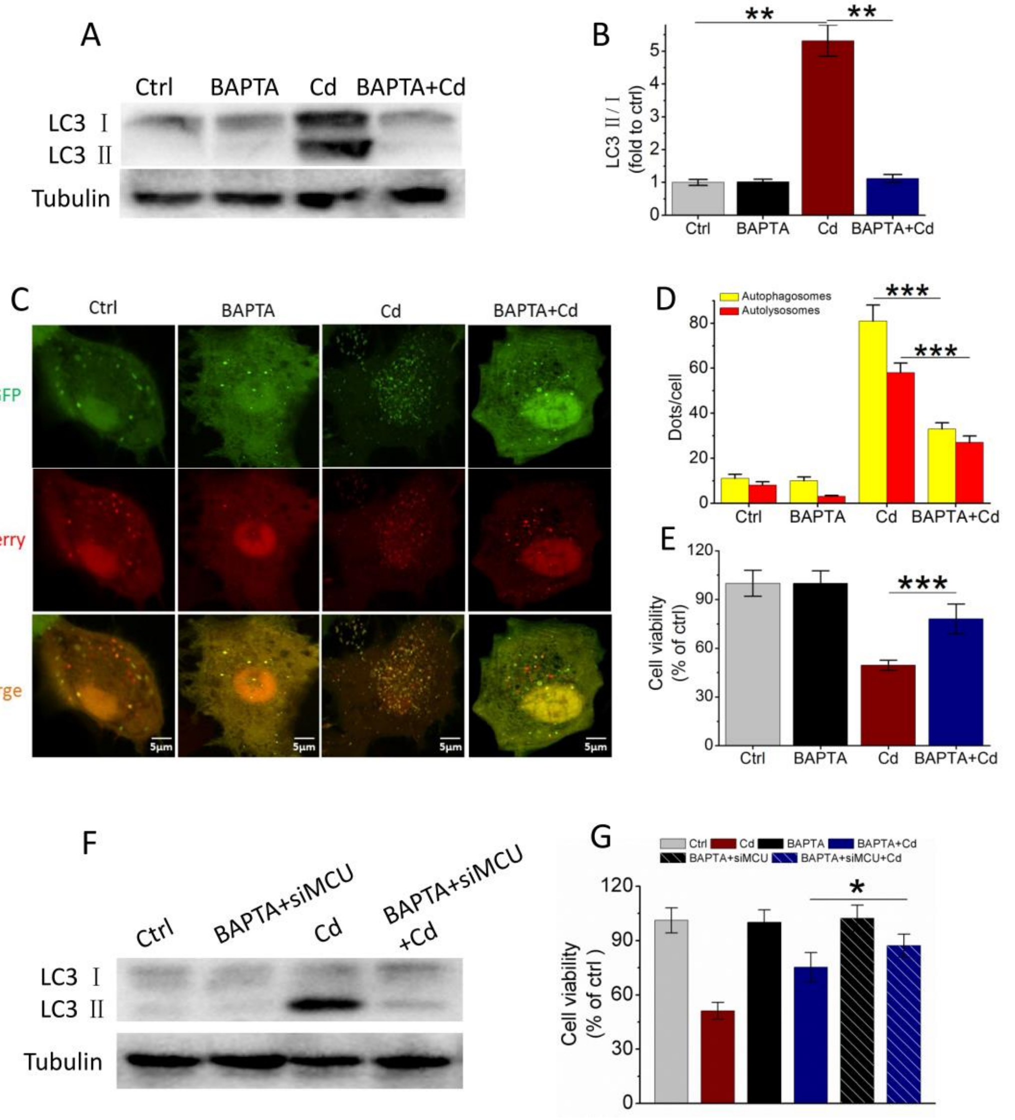

Supplementary figure 11: Effects of BAPTA on autophagic cell death induced by Cd. A) Immunoblots of LC3 Ⅱ/Ⅰ from cell lysates treated with BAPTA or/and Cd. Tubulin served as the loading control. B) Quantification of LC3 Ⅱ/Ⅰ from (A), $n = 3$. C) Confocal images for analysing autophagic flux in cells treated with BAPTA or/and Cd. D) Quantification of autophagosomes (yellow) and autolysosomes (red), $n = 20$. E) Cell viability change after BAPTA pretreatment before Cd ($n = 5$). F) Immunoblots of LC3 Ⅱ/Ⅰ from cell lysates of indicated groups. G) MCU deficiency further improved BAPTA-elevated cell viability after Cd exposure ($n = 5$). *$p < 0.05$, **$p < 0.01$, ***$p < 0.001$. ns, no significance.

Figure S12

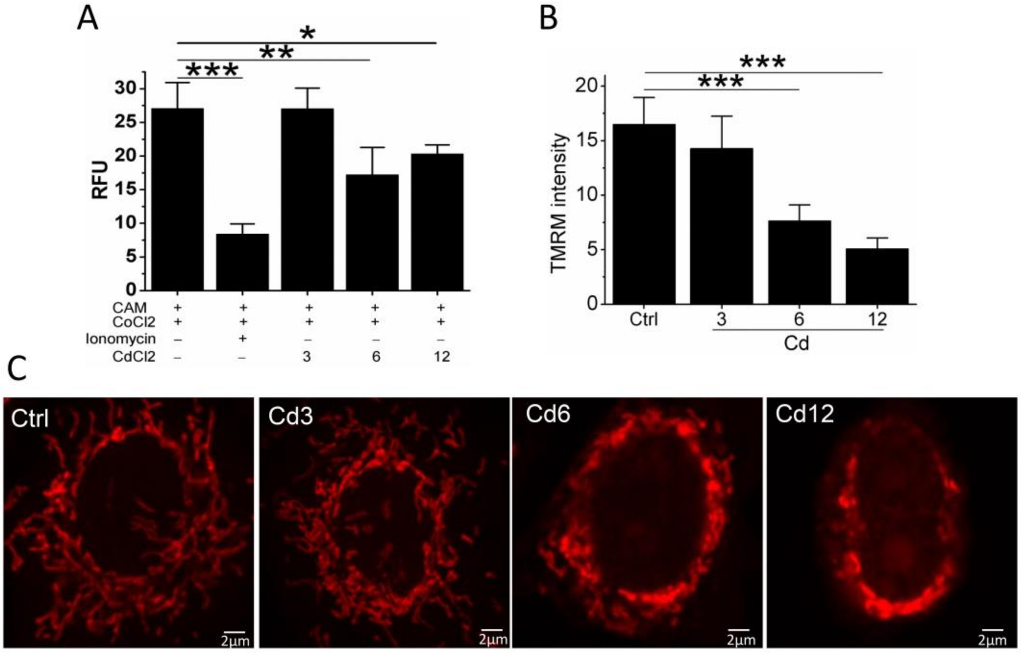

Supplementary figure 12: Cd promoted opening of mitochondrial permeability transition pore. A) mPTP assay kit was used to evaluate the opening of mPTP. CAM (calcein acetoxymethyl ester) could penetrate into cytoplasm and other subcellular organelles (mainly mitochondria) and be hydrolyzed to calcein. Calcein showed bright green fluorescence that could be quenched by CoCl2. The more mPTP open, the lower mitochondrial fluorescence intensity. Cells were exposed to 3, 6 and 12 μM Cd for 6 hours, and the fluorescence was detected and quantified after CAM and CoCl2 addition (n = 5). B) Mitochondrial membrane potential evaluated by TMRM. Cells were exposed to 3, 6 and 12 μM Cd for 12 hours and the fluorescence intensity was detected by microplate reader at 550/575 nm (n = 5). C) Evaluation of mitochondrial morphology after 3, 6 and 12 μM Cd exposure for 12 hours. Mitochondria were stained by MitoTracker Red (200 nM). Representative confocal images were given. *$p < 0.05$, **$p < 0.01$, ***$p < 0.001$.

Figure S13

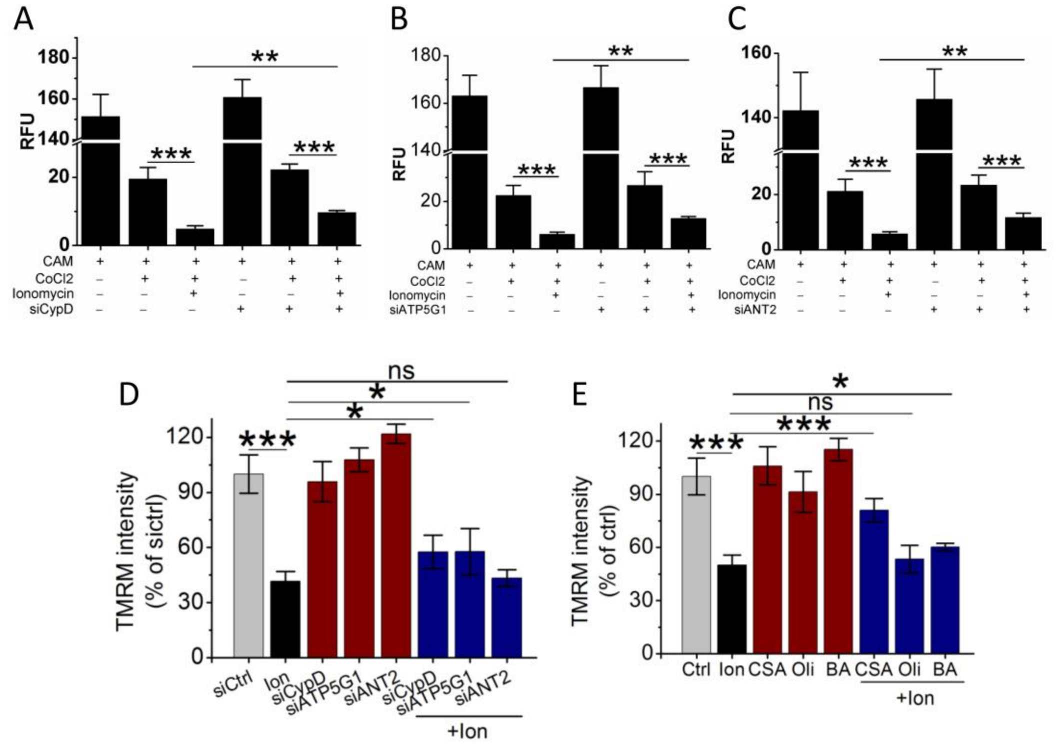

Supplementary figure 13: Effects of CypD, ATP5G1 and ANT2 on mPTP opening. A to C) Genetic depletion of CypD (A), ATP5G1 (B) and ANT2 (C) inhibited mPTP opening (n = 5). HepG2 cells were transfected with indicated siRNA for 48 hours before addition of indicated detection agents using mPTP assay kit as described previously. The calcein RFU (relative fluorescence unit) was measured by microplate reader. Ionomycin served as the positive drug quenching mitochondrial calcein fluorescence. D) mPTP opening assessed by mitochondrial membrane potential alteration (n = 5). HepG2 cells were tansfected with indicated siRNA for 48 hours, and the ionomycin was added before TMRM stain. The TMRM intensity was detected by microplate reader at 550/575 nm (ex/em). E) CSA (CypD inhibitor), oligomycin (ATP synthase inhibitor) and bongkreic acid (ANT2 inhibitor) were utilized for assessment of mitochondrial membrane potential by TMRM (n = 5). These inhibitors (10 µM) were added into cells 2 hours before ionomycin (10 µM) addition. Ion, ionomycin. Oli, oligomycin. BA, bongkreic acid. *$p < 0.05$, **$p < 0.01$, ***$p < 0.001$. ns, no significance.

Figure S14

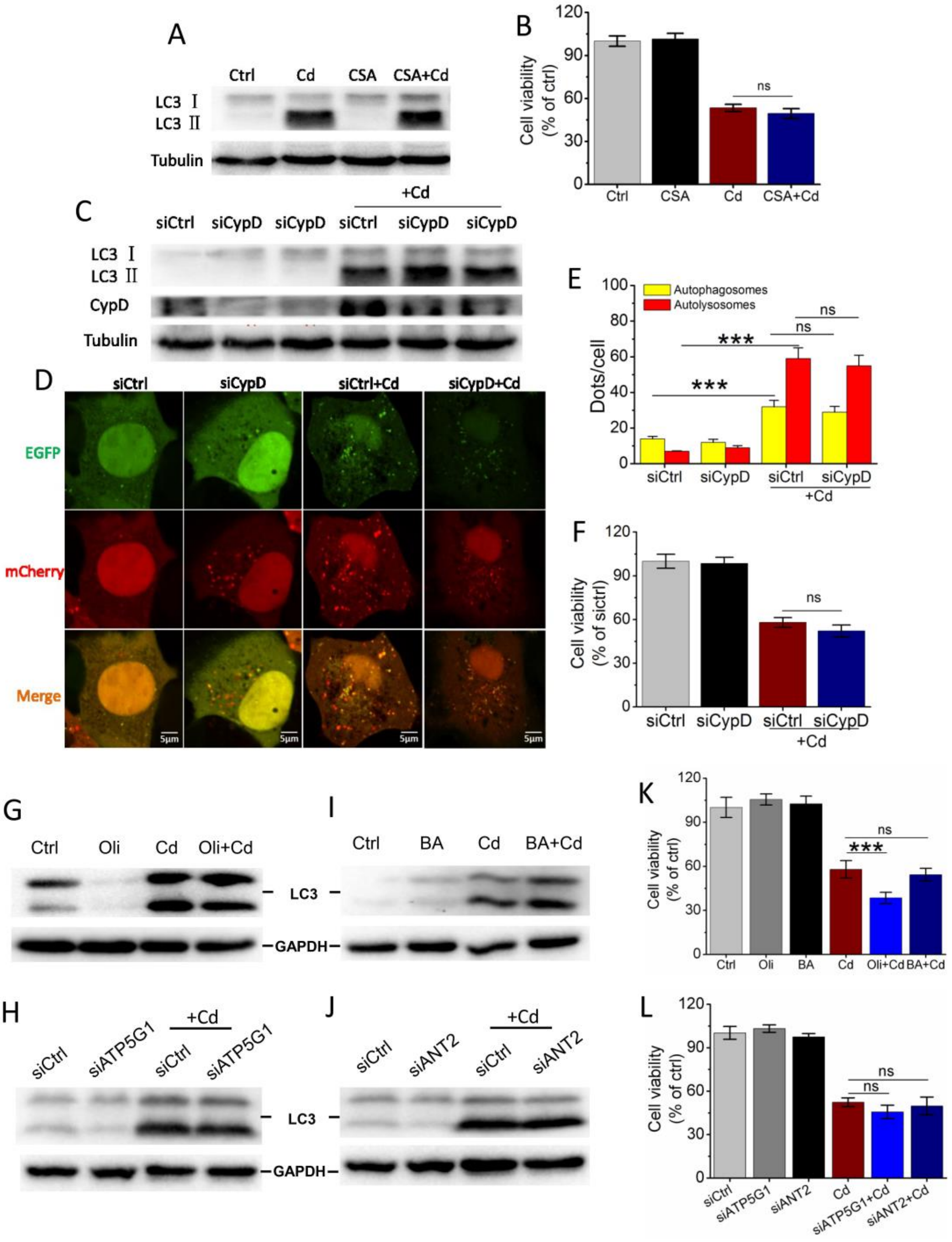

Supplementary figure 14: Inhibition of MPTP failed to alleviate excessive autophagy and to improve cell viability. A) Immunoblots of LC3 II/I in cell lysates treated with CSA or/and Cd. B) Cell viability after CSA or/and Cd treatment (n = 5). C) Immunoblots of LC3 II/I in cell lysates treated with siCypD or/and Cd. D and E) Effects of siCypD on overactive autophagic flux. (D) Autophagic flux evaluation by confocal microscopy. (E) Quantification

of autophagosomes and autolysosomes (*n* = 20). F) Effect of siCypD on cell viability (*n* = 5). G and H) LC3 Ⅱ/Ⅰ expression change analyzed by immunoblotting after oligomycin (G) and siATP5G1 (H) treatment. I and J) LC3 Ⅱ/Ⅰ expression change analyzed by immunoblotting after bongkreic acid (I) and siANT2 (J) treatment. K) Effects of oligomycin and bongkreic acid on cell viability after Cd exposure (*n* = 5). L) Effects of siATP5G1 and siANT2 on cell viability after Cd exposure (*n* = 5). Oli, oligomycin. BA, bongkreic acid. ***p < 0.001. ns, no significance.

Figure S15

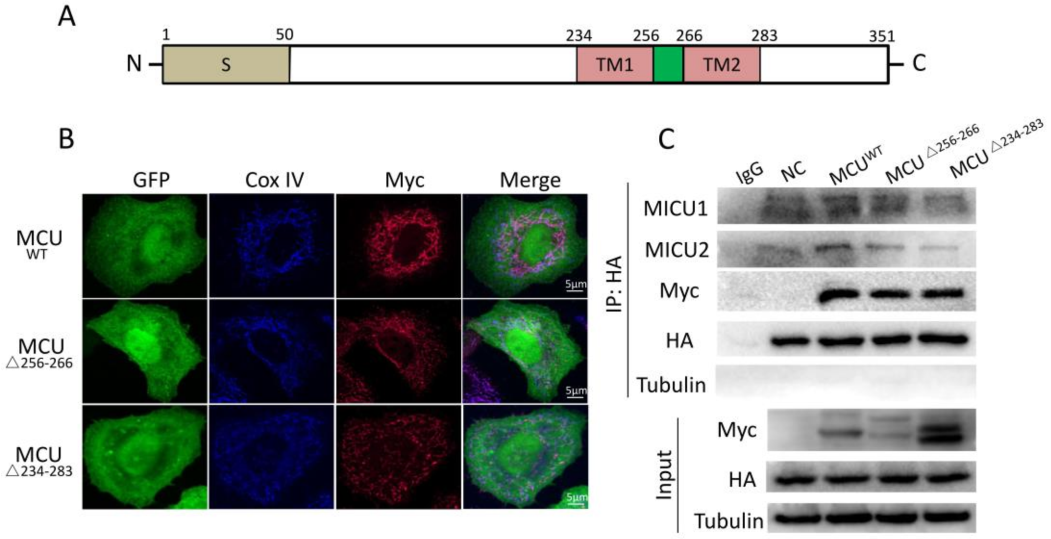

Supplementary figure 15: MCU physically interacted with VDAC1. A) Schematic diagram of MCU mutants. The green color indicated the intermembrane space (IMS) domain, and the pink color indicated the transmembrane (TM) domain. Three MCU mutants, MCU^{WT}, $MCU^{\Delta 256\text{-}266}$ and $MCU^{\Delta 234\text{-}283}$ labeled with myc-tag were constructed (GeneChem). WT, wide type; Δ, deletion; N, N-terminal domain; C, C-terminal domain. B) The mitochondrial translocation of MCU was not affected after deletion of aa256-266 or aa234-283. Confocal images were taken from HepG2 cells transfected with indicated constructs. Cox Ⅳ was stained to label mitochondria. C) The interaction between MCU and VDAC1 still existed after deletion of the IMS and TM domain. The CoIP assay was performed in HepG2 cells overexpressing HA-VDAC1 that was further transected with indicated MCU mutants.

Figure S16

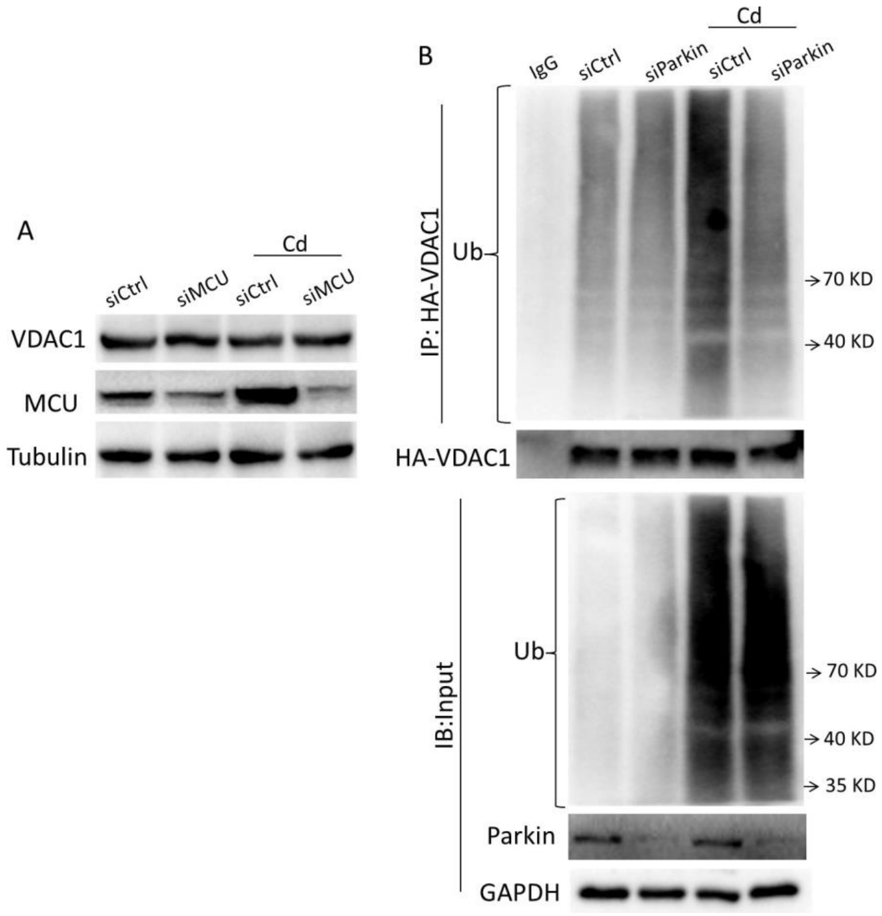

Supplementary figure 16: The effect of MCU on VDAC1 expression and the effect of Parkin on VDAC1 ubiquitination. A) Knockdown of MCU didn't affect VDAC1 expression. HepG2 cells were transfected with siMCU for 48 hours before treated with 12 μM Cd for 12 hours. The lysates were analyzed by immunoblot. B) Parkin mediates VDAC1 ubiquitination. PINK1-mediated mitophagy needed involvement of Parkin. The siParkin was transfected into HepG2 cells for 48 hours before exposure to 12 μM Cd for 12 hours, and the total cell lysates were harvested for further immunoblot analysis. The bottom panel indicated the total protein ubiquitination, and the upper panel showed the ubiquitination of VDAC1.

Figure S17

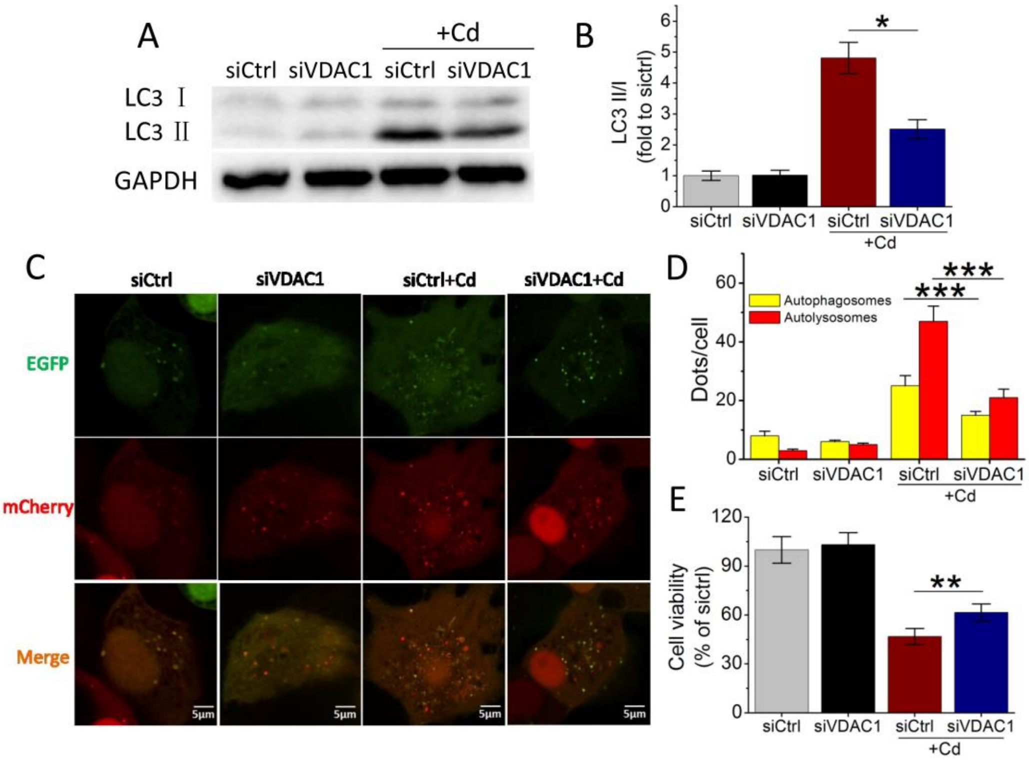

Supplementary figure 17: VDAC1 deficiency attenuated autophagic cell death caused by Cd. A) Immunoblots of LC3 Ⅱ/Ⅰ from lysates pretreated with siVDAC1 for 48 hours before exposure to 12 μM Cd for 12 hours. B) Quantification of LC3 Ⅱ/Ⅰ from (A), $n = 3$. C) Alternation of autophagic flux after siVDAC1 treatment. Cells were treated as previously described. D) Quantification of autophagosomes and autolysomes ($n = 20$). E) Cell viability after siVDAC1 treatment ($n = 5$). $^*p < 0.05$, $^{**}p < 0.01$, $^{***}p < 0.001$.

Figure S18

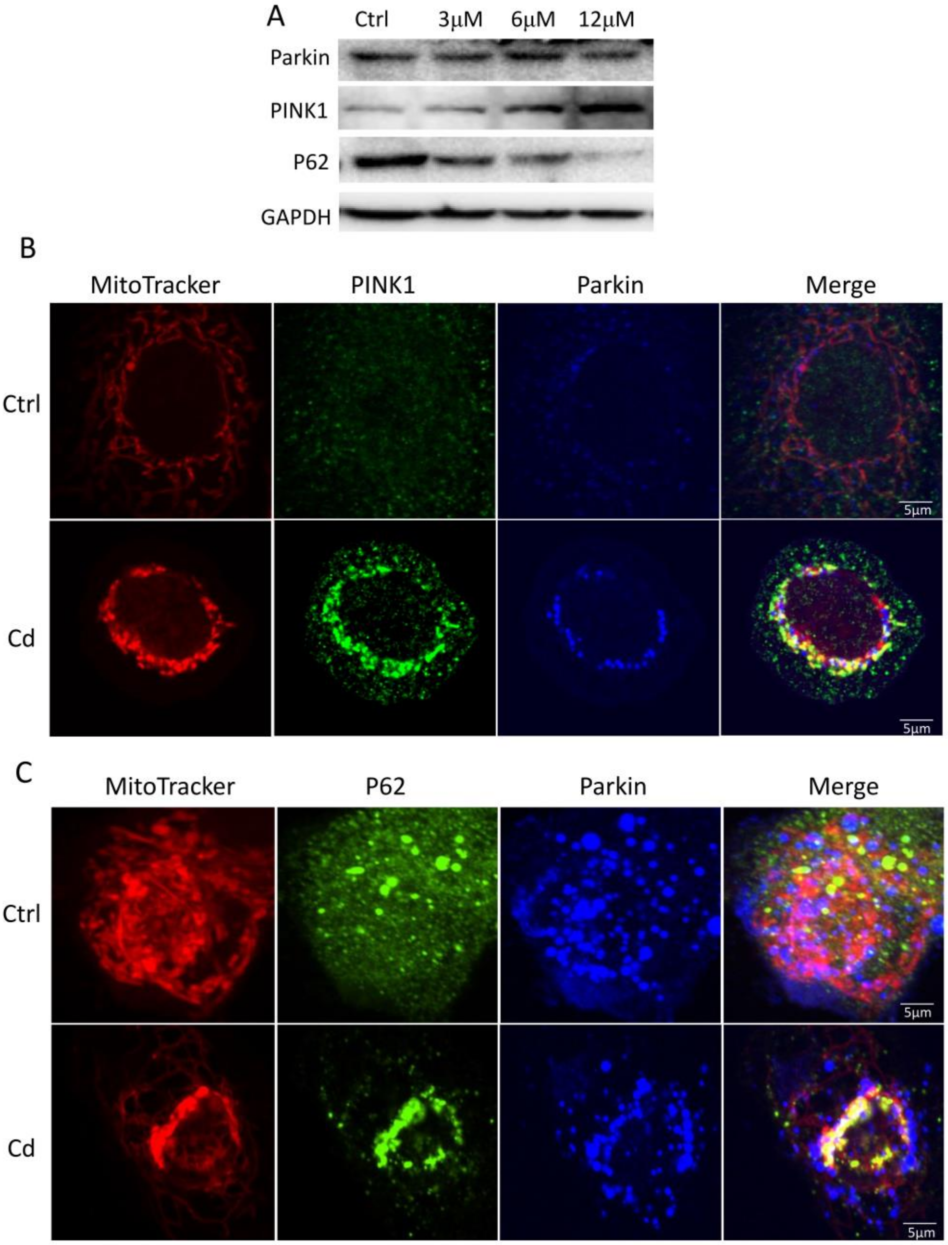

Supplementary figure 18: Cd-induced expression alternation of Parkin, PINK1 and p62, and their mitochondrial translocation. A) Immunoblots of Parkin, PINK1, and p62 after various dosage of Cd exposure. GAPDH was used as loading control. B) Mitochondrial translocation of Parkin and PINK1. Cells were exposed to 12 μM Cd for 12 hours, and then stained by MitoTracker Red, fixed by 4% paraformaldehyde, probed with two indicated primary antibodies and secondary fluorescent antibodies. The images were captured by confocal microscopy at 561nm (red), 488nm (green) and 405nm (blue) excitation, respectively. C) Mitochondrial translocation of Parkin and p62.

Figure S19

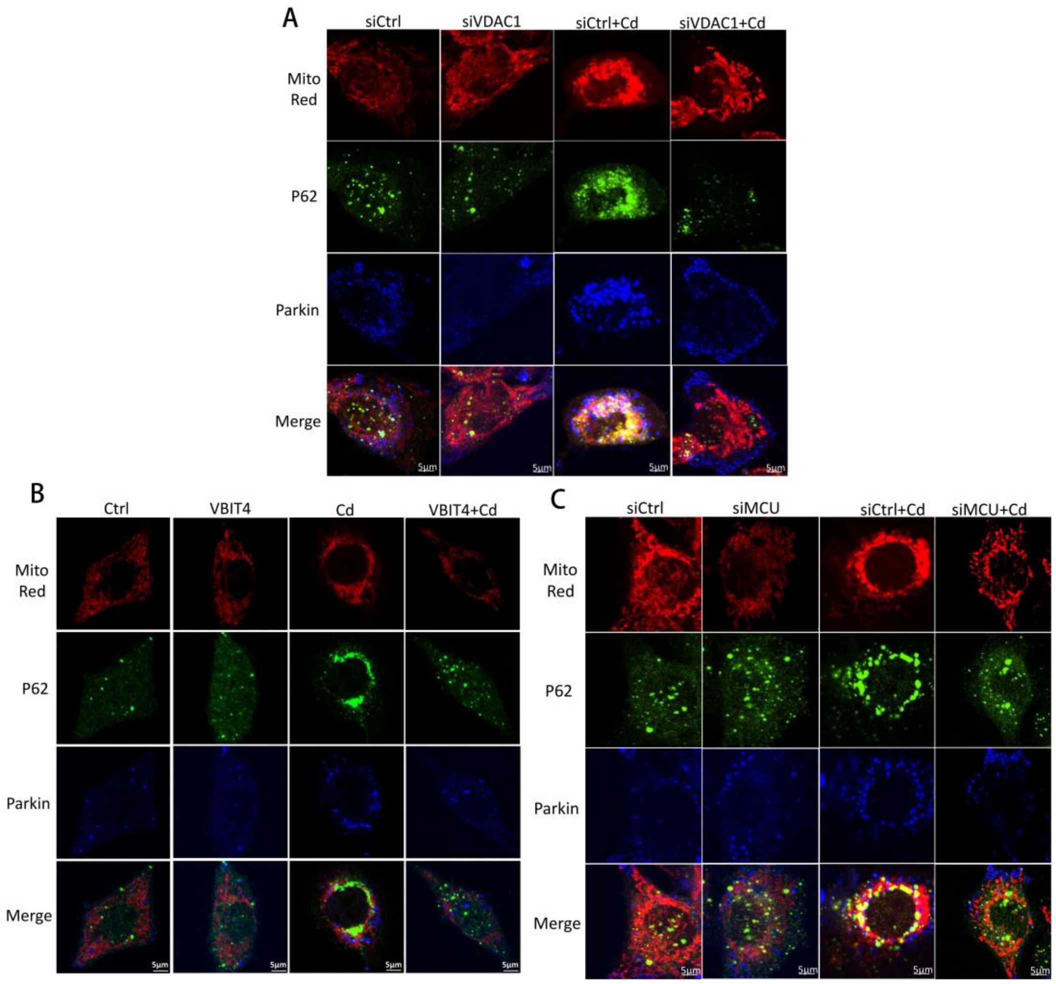

Supplementary figure 19: The effects of siVDAC1, VBIT4 and siMCU on mitochondrial translocation of Parkin and p62. A to C) Confocal images indicating mitochondrial translocation of Parkin and p62 in cells pretreated with siVDAC1 (A), VBIT4 (B) and siMCU (C) before Cd exposure. The Parkin and p62 primary antibodies were mixed and incubated together before probing with two different secondary fluorescent antibodies. The representative images are shown.

Figure S20

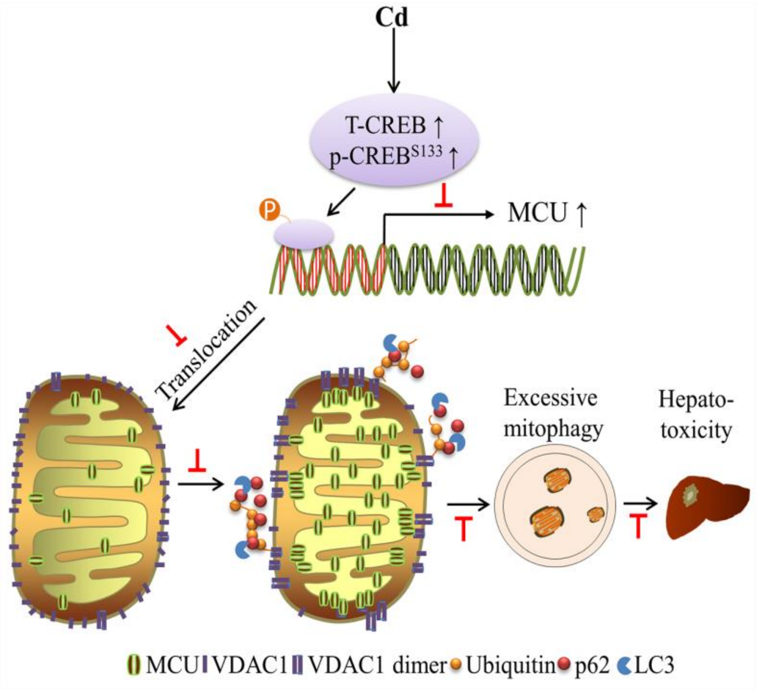

Supplementary figure 20: Schematic illustration showing that Cd promoted MCU overexpression by elevated phosphorylation of CREB and then increased the dimerization and ubiquitination of VDAC1 by their interaction, and then increased mitochondrial recruitments of p62 and LC3, leading to excessive mitophagy and hepatotoxicity. The ubiquitination of VDAC1 was mediated by Parkin and PINK1 that translocated into mitochondria.

Table S1. Autophagy related gene (ATG) readcount result from RNA-seq.

ATG gene	Con1	Con2	Con3	Con4	Cd1	Cd2	Cd3	Cd4
ATG2A*	708	761	619	860	825	991	813	937
ATG2B	481	586	448	574	365	213	349	343
ATG3	370	408	313	365	487	329	543	491
ATG4A*	51	68	53	51	155	133	157	146
ATG4B	2750	3010	2374	2441	2789	3191	2970	2997
ATG4C	172	164	145	153	136	67	171	125
ATG4D*	250	253	203	269	335	492	339	386
ATG5	439	467	370	411	445	265	521	523
ATG7*	206	236	185	241	266	274	255	262
ATG9A	1464	1571	1334	1466	1381	1843	1431	1356
ATG9B	15	11	9	9	10	8	14	9
ATG10	72	85	65	95	53	47	86	92
ATG12	618	643	570	570	548	314	606	599
ATG12P1	5	12	11	7	10	7	16	6
ATG13	1239	1280	1050	1323	1058	1312	995	1173
ATG14	316	362	275	344	227	164	260	274
ATG16L1*	770	892	695	811	1591	1852	1685	1629
ATG16L2	463	548	411	352	389	231	373	473
ATG101*	519	515	412	626	807	1113	887	996

The left bold gene name indicated an increase trend or significant increase (*$p < 0.05$, compared with control group) in gene expression after Cd exposure.

Table S2. Readcount result of mcu and its subunits from RNA-seq.

Gene	**Con1**	**Con2**	**Con3**	**Con4**	**Cd1**	**Cd2**	**Cd3**	**Cd4**
*MCU**	430	510	408	497	575	555	563	669
*EMRE**	5141	5262	4096	4647	5305	7401	5937	6750
MCUb	66	79	59	92	88	59	99	76
*MICU1**	1322	1333	963	1247	674	741	684	663
*MICU2**	538	576	458	585	884	753	875	821

*$p < 0.05$, compared to control group.

Table S3. siRNA and oligos.

1. siRNA

siRNA	Target sequence (5'→3')
Homo-siATG5	CAACTTGTTTCACGCTATA
Homo-siPINK1	GGACGCTGTTCCTCGTTAT
Homo-siMCU	GTACGAATTGAGATTAGCA
Homo-siCREB	GCTCGAGAGTGTCGTAGAA
Homo-siCypD	GAATCTTTCGGCTCTAAGA
Homo-siATP5G1	GTGTCTGCCTCCTTCTTGA
Homo-siANT2	GCGGAAGATTGCTCGTGAT
Homo-siVDAC1	GCTGCGACATGGATTTCGA
Homo-siParkin	AGTCGGAACATCACTTGCA

2. Oligos

Gene	Oligos (5'→3')
MCU(genotyping)	F2: GGAGTTAAGTCATGAGCTGCTAT<br>R2: CTGGCTTAGTTGGCAGAGTTC<br><br>F2: GGAGTTAAGTCATGAGCTGCTAT<br>V76R: CCAATAAACCCTCTTGCAGTTGC
Homo-MCU	F: TCCAGAAGCCAGAGACAGAC<br>R: TGTCGGAGAGGCAGATGTAC
Homo-Gapdh	F: GGAGCGAGATCCCTCCAAAAT<br>R: GGCTGTTGTCATACTTCTCATGG

Table S4. Methods for predicating the binding sites of CREB on *mcu* promoter.

Location	Sequence	Method
-495-484	CTCCGT GATGTA	http://consite.genereg.net/. The *mcu* promoter sequence (2000bp) was input, and homo CREB was chosen as a transcription factor. According to the result, the CTCCGTGATGTA sequence was the only potential binding site at "+" strand with high score.
-599-594	ACGTCA	https://biogridlasagna.engr.uconn.edu/lasagna_search/. LASAGNA-aligned models were chosen, followed by choosing homo CREB as transcription factor, *mcu* promoter as binding sequence. The results indicated that both ACGTCAGTT and ACGTCATTT were candidate binding sites of CREB on *mcu* promoter. However, the former site has a higher evaluation score, and was selected as the possible binding site.
-1630-1623	TGAG GTCT	https://jaspar.genereg.net/. This binding site was not predicated by upper two methods. By visiting this website, we identified 7 potential CREB binding sites at "+" strand of *mcu* promoter (2000bp), including the "-495-484" sequence. As one of the 7 binding sites, TGAGGTCT has a highest score of 8.13 at -1000-2000 region, so it was chosen as a possible potential binding site for CREB.

Table S5. Location of three mutation sites on *mcu* promoter.

***mcu* promoter sequence**
**(-2000)**<br>**T**CTTTTGCTAATGCATATGAGTAAATCTCACTGCAGGATGCACATCTAATTTTCATGCTTGG<br>AAAAATAGAGTAAAGGCAATTCTAGTAAAATTTCTTAAAGTGGAAAGTGGAAATGGAGCA<br>ATCCTGATTCATAGCTAGATCTGGTGACAGCACAGAAAATAAAAACAAAACTAAAATCCAT<br>GGTGATCTCTGTAAGAAGGCATGAAGGGGTGAGAGAGAAAATGGCACAGCATGGGTAAAT<br>ACATCAAATGCAGTTGGGATAAATATAGATGGGTGGAAAATAATTGTAAGGGAAATTGTTC<br>CAGGAATGTCATGGATTTCCAACTAGGGGGATTACTGTGTGCAGCTGTTGAATGGGGGCTC<br>**-1630** **-1623**<br>ATTCTTGAGGTCTCAAGTTCCACCTGTTGGGCAATAAAGCCTTTTGAGTGAGTGACATGAT<br>ATCTACCCCGGAGAAGCCAATCATCTCACTGTTTTGGTGTAGGTGGGGCATAAGGATTAAA<br>CAAACAAAAAACTTCCACGTCATTTTTTAGCTATTCTTGGCCAAGAAGGTACCATTTCAGT<br>CAAACTTTAAAGAATATTCTTAGAATAAGTGATAACATTTTCTTCGTATTTATGTGTGTGTTT<br>TGTTTTTGTTTTAAATTTATCATCTAAGGCTACACTACAAAGATGTTTTCCAAAATTTAAAAT<br>AATGCTTGTATTTGCTTCCCAAACTATTTTCCTGCTCCAGATTTTAAAAAGAGGTGTGAGAA<br>TTTAATCAGCTCATAATAATAAACAGCTAGCAAGGAAATACTCTTCTAAGGAAATTCAGGA<br>CCCAAAATCTTTCTTAGGCAGTATTCAAGGAGCCCTCATTGTGAGTTTAAGTGGAAAGAAG<br>TGTTTTTCAAGAGAAAGTGTTTGTTATTCCAAAGCGTATTCTGATAACTATTTCGGCAAAAT<br>TATGAAGCATCATTAAAATAGCCTTTTACATGAATTTTTTTTTTTTTTTTTGAGATGGAGTCT<br>TGCTGTGTCGCCCAGGCTGGAGTGCAATGGCCCGATCTCGGTTCACTGCAAGCTCCGTCT<br>CCCGGGTTCACACCATTCTCCTGCCTCAGCCTCCGGAATAGCTGGGACTATAAGCGCCCGC<br>CACCACGCCTGGCTAATTTTTTGTATTTTTAGTACAGATGGGGTTTCACCGTGTTAGCCAGG<br>ATGGTCTCGATCTCCTGACCTCGTGATCCGCCCGCCTCTGCCTCCCAAAGTGCTGGGATTA<br>CAGGCGTGAGCCACCGCGCCCGGCCTTACATGATTTTTTATTACGTTATTAATGTGTTCTGT<br>TTCTGTAAATGCACACAAAGTCAAATATGATAAAATAGTGCTGTTTCTGGAGAGTGTGAGA<br>**-599** **-594**<br>ATATCCGTATAAAGGTGCTGTAAAAATGAATTGGGTATTTGAGATGTATACACGTCAGTTAA<br>TTCACTCTGAAGGCATGTACTCATTCTGGGACTTTGATATTGTAATCGTTCTACCTCTCCTAT<br>**-495** **-484**<br>ATTATGCTACGCATCACAGTCATTAGATCACTCCGTGATGTAGAGTTAATTTCAGCATATCTA<br>AATATTATCAGTTATGAATGATAATTCTAGGCGTGTAGTTGAGAGTTACAGCATACACCGTT<br>AATGGTTATTAATTTGGTGCCTATTTTGCCAACTTAGAACACTCACTTAGAACTCAACTTCG<br>AACAAAGCTTTTCACAAATATCTATATCCCCCGTTACTCTTATCATAGCTCTATTGTTGGTGA<br>AGATTAGCCGAACTGAACATGCCATCTCTATTGCTGTTATTTTCACGGGTTTGGGTGTCAAT<br>TATGGGTTCCCTAGAATTTGCCCGTAATTGACTATGTCCCACTATAGGTAACTCTGGAAGGG<br>ACTGGCAGCTTCCAGTGTGGTTCTCCCGCCTGTCGATTGGACCTTGCAGGAAGCCCCGCC<br>TCATCAACTCAGTCAAGGGCTTTAGTTGGCCCGGCGTCGGTGAGTGGCGGGTCTTAGCTC<br>CGCCCTGGCCGTCCGCTGCGCTGCCTGGGT**C**G(TSS)
